# Conformational deformation of a multi-jointed elastic loop

**DOI:** 10.1038/s41598-022-24355-7

**Published:** 2022-11-21

**Authors:** Hiro Tanaka, Yuji Seki, Shohei Ueno, Yoji Shibutani

**Affiliations:** 1 Department of Mechanical Engineering, Osaka University, 2-1 Yamadaoka, Suita, Osaka 565-0871 Japan; 2 Nanotechnology Program, VNU Vietnam Japan University, Luu Huu Phuoc Street, My Dinh 1 Ward, Nam Tu Liem District, Hanoi, Vietnam

**Keywords:** Mechanical engineering, Applied physics, Mechanical properties

## Abstract

A new class of deformation is presented for a planar loop structure made up of slender elastic bodies and joints. In demonstrating the circumferential shortening of the multi-jointed elastic loop, diverse three-dimensional (3D) deformations emerge through piecewise deflections and discrete rotations. These 3D morphologies correspond to conformations of molecular ring systems. Through image processing, the 3D reconstructions of the deformed structures are characterized by number, geometry, and initial imperfections of the body segments. We elucidate from measurements that the conformational deformation without self-stress results from a cyclical assembly of compressive bending of elastic bodies with high shear rigidity. The mechanical insights gained may apply in controlling the polymorphism exhibited by the cyclical structures across scales.

## Introduction

Slender structures, for which the transverse sectional dimension is much smaller than the longitudinal sectional dimension, are ubiquitous in many disciplines. They are found everywhere at different length scales from sub-oceanic cables to human-sized rods and ropes, to plant and animal microstructural fabrics, to molecular pillars and chains such as carbon nanotubes and double-stranded deoxyribonucleic acids (DNAs). Their unique large deformability have received attention from the scientific community including the field of theoretical and applied mechanics, and even at present the problems associated with the intricate deformation into three-dimensional (3D) configurations are being tackled in areas such as the compressive buckling from 2D into 3D architecture^1–3^, the coiling of ealstic filaments deployed on substrate^4,5^, the mechanics of knots^6,7^, and the 3D growing rods^8–12^.

The deformation of a slender elastic body is concisely described by the elastic rod model represented by a single arc-length parameter prescribing the center line^13,14^. Depending on the elastic rod, there are four types of deformations, i.e., stretching/compression, shearing, bending, and twisting, which are paired with the axial and shear forces and the bending and torsion moments induced inside the rod. Hockling of stretched and twisted cables is a typical example of large 3D deformations of inextensible elastic rods without shearing, collectively named *Kirchhoff’s rods*. The terminal torques and low tensions applied to an initially straight configuration induce helical buckling, whereby the deformation mode transitions from torsion to 3D deflection^15–17^. After a helix begins to form locally, more and more twisting yields a loop of helices as a self-contacting formation perpendicular to the longitudinal axis. This writhing leads to a final phase called a *plectoneme*; e.g., a plectonemic phase emerges in supercoiled helixes of DNA^18–21^.

Non-negligible stretching and shearing cause, after uniform helical buckling, another type of deformation that features self-contact, known as a *solenoid*^22^, which involves shortest-wavelength writhing in the longitudinal direction. The uniform helical formation and the post-buckling localization, including the plectonemic or solenoid phase, can be simulated using elastic rod theory and its extension. The extended theory describes the local manner of deformation through axial extension and shear as well as that of bending and torsion^23,24^. Although understanding this hockling phenomena is of significance from the viewpoint of the 3D deformation of higher-order structures, the resulting coiled morphology is ill-reproduced, in general, given the specific control parameters such as the external action of terminal torsion and stretching.

A control of the diverse structural morphologies undergoing large deformation is of interest to the various fields as illustrated previously. In this context, we focus on modeling that reduces the limited degrees of freedom in deformation to achieve a controllable system for the 3D morphology. We present another concept of a slender structure subjected to conventional contraction forces that is capable of being deformed into countable 3D patterns. The concept concerns the tangle mobility of interconnected pivotal segments, collectively referred to as a *tangle model*^25^. The tangle model comprises a number of rigid elbowed bodies, linked in a loop by revolute hinges. The possible morphology corresponds to the conformation of a molecular ring system, which is described by the Dreiding stereo model or through conformational analyses^26,27^.

Our main structural idea is to replace all the rigid equilateral components in a tangle model with soft tubular bodies. These elastic bodies are intersected via joints in a loop configuration, and tuning of the number of bodies and joints can control the degrees of freedom for each deformation. In the design of our proposed structure, we select bellowed tubing for the geometry of the elastic body. Bellows are an ancient mechanical component that have been used where compression and/or flexion is required. Their fundamental mechanical properties have been established from modelling their elasto-plasticity to beam modelling using finite-element methods^28–30^.

With deformable loops made of a finite number of these elastic bodies and joints, we examined the circumferential shortening of each loop, which is equivalent to applying resultant forces directed towards the center. Following an analysis of the conformational deformations accompanying the piecewise deflections and discrete rotations, we report the manner of the various morphologies, which depends on structural factors, specifically, the number of bodies, the stiffness of stretching, bending, and twisting, and initial imperfections.

## Results

**Figure 1:**
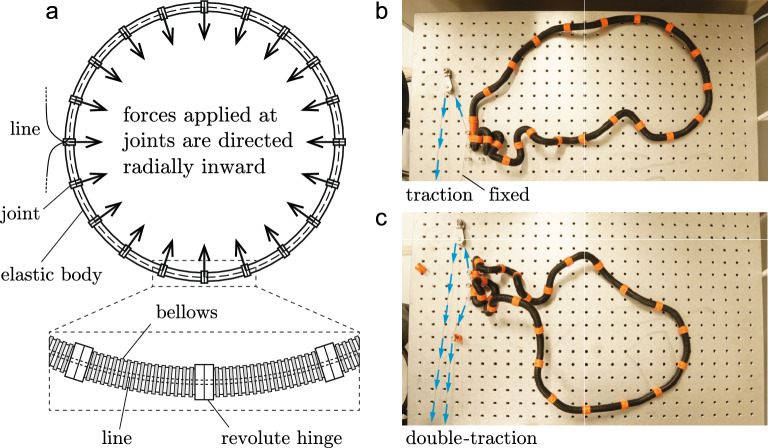
(**a**) Schematic of an ideal multi-jointed loop and a magnified view of the modules comprising elastic bodies and joints; its traction line passes through the midline of the loop. Traction yields concentrated forces acting toward the center. (**b**,**c**) Deformed morphology for the fabricated structure with $n=23$: (**b**) single-traction test and (**c**) double-traction test. The blue arrows indicate the tractional directions of the single line made of steel wire.

The proposed structure forms a loop, assembled alternately with equilateral elastic bodies and joints. Figure 1a illustrates the main structure and its modules in magnification. A module comprises a bellows tube (body) connected with revolute hinges (joints) at both ends. A single compliant line passes along the midline of the multiple modules (the dashed line in Fig. 1a). The line is not closed and both ends are connected to an external device. Note that a revolute rotation on a joint may occur as a rotation of the revolute hinge itself or a change in the dihedral angle formed by the adjacent tubes bent in different directions. We refer the latter as a *pseudo-rotation*.

If the line inside the structure is shortened, the multi-jointed loop is compressed circumferentially. The resultant of the corresponding compressive force vectors acting on a joint is a force concentrated toward the center and all the point-symmetric forces on the circle are regarded as a cohesive attraction. The number of joints equals the number of point forces; the joints play a role in allocating points of application on the midline of the loop.

This loading can be applied in a traction test in which one reels off the line at a single side or both sides using a mechanical device such as a winch; hereafter, such tests are referred to as *single-traction/double-traction tests*. In the deformed configuration with bending, the direction of each force is shifted toward that of the local radius of curvature.

Figure 1b,c present the morphologies obtained in single- and double-traction tests for $n=23$, where *n* is the number of bodies and $n=23$  is the largest size possible for an introductory experiment. Details of the structure and experiments are described in Supplementary Information S1.1 and S2.1, and two animations are provided as Supplementary Movies S1 and S2 (see Supplementary Information). In each test, the structure exhibits 3D deformation induced by the bending of bodies and rotating of joints. These 3D coiled behaviors are localized in the traction direction(s). In other words, the deformation is concentrated at the terminal side(s) in the single-traction test (Fig. 1b) and double-traction test (Fig. 1c). The localized morphology of (b) is similar to a solenoid-like pattern observed for a clamped filament subjected to tensile and torsion forces at a free end^22,24^.

**Figure 2:**
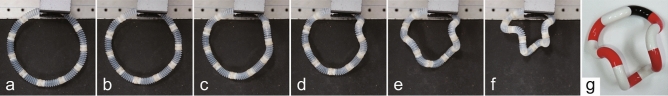
(**a**)–(**f**) Snapshots of the deformation of a bellows-type structure with $n=10$ in the double-traction test (see also Supplementary Movie S3). (**g**) Similar form of a tangle model with $n=11$, where the black body placed at the top is the complement of a space in the experiment.

To investigate how the 3D deformation is affected by the geometry of elastic bodies, we performed double-traction tests for two different shapes, specifically, bellows and straight tubes. For the additional tests, we prepared small loops with $n=10$. The geometry and materials of the components are described in Supplementary Information S1.2. The experimental results show that the bellows-type structure exhibited 3D deformation whereas the straight-type structure maintained its in-plane shrinkage (see Supplementary Information S2.2 and Movies S3 and S4). A sequence of the 3D deformation in Fig. 2a–f shows that the structure shrinks uniformly because, in the early stages, it is confined to a two-dimensional plane. The structure then begins to form a 3D configuration through continuum bending and discrete rotation. The bending deformation proceeds to nearly a right angle in each body. The morphology in (f) is similar to that of a tangle model made of 11 rigid bodies having $90^{\circ }$ curvature (Fig. 2g), and it can thus be linked to a conformation similar to that of a molecular ring system. During the conformational deformation (Fig. 2c–f), the pseudo-rotations were dominant because little variation was observed in the planar rotation between a pair of joint surfaces.

In the unloading test (Supplementary Movie S5), the conformational deformation turned out to be a viscoelastic-like behavior because the bellows-type structure underwent reverse deformation to the original state. Note that the reversible change ceased mainly through friction with the substrate, but a small plastic strain remained in bending.

These traction tests reveal that the pseudo-rotations of joints occur discretely as conformational deformation only in the bellows-type body and that the straight-type body shrinks continuously and there is no conformation. To explain why conformation depends on geometry, we assess the rigidity for straight and bellows tubes. We introduce the ratio of torsional to bending stiffness of a uniform slender body,

1 $$\begin{aligned} \gamma \equiv \dfrac{GI_{\textrm{p}}}{EI}, \end{aligned}$$

where *E* and *G* denote the Young and shear moduli of the material, and *I* and $I_{\textrm{p}}$ the second moment and second polar moment of the area. For a hollow circular cross-section with an average radius of $r_{\textrm{m}}$ and small thickness of *t*, $\gamma = 2G/E$ because $I_{\textrm{p}}=2I$, resulting from $I \simeq \pi r_{\textrm{m}}^3 t$ and $I_{\textrm{p}}\simeq 2\pi r_{\textrm{m}}^3 t$.

The effective bending and torsional stiffness of the bellows with a length of *L* are approximately expressed as

2 $$\begin{aligned} E I^*\simeq \dfrac{r^2_{\textrm{m}}k_x L}{2},\quad GI_{\textrm{p}}^*\simeq 2\pi G r^3_{\textrm{m}}t\left( \dfrac{L}{L_0}\right), \end{aligned}$$

where $k_x$ and $L_0$ are respectively the spring constant and total corrugation length of the bellows^30^. The former refers to the effective axial rigidity of the thin-walled tube analogy; i.e., $k_x = EA/L$, where $A=2\pi r_{\textrm{m}}t$. For the small member, the bellows tube has nine inclined corrugations per member length *L*, and the geometry is simplified as a sawtooth waveform so that $L_0$ may be calculated approximately (see Supplementary Information S1.2).

Considering $\gamma$ of the straight tube, which comprises the same material as the bellows, we derive the relationship

3 $$\begin{aligned} \gamma ^*= \dfrac{GI^*_{\textrm{p}}}{EI^*} \simeq \dfrac{L}{L_0}\left( \dfrac{EA/L}{k_x}\right) \gamma . \end{aligned}$$

Equation (3) allows us to estimate that $\gamma ^*\gg \gamma$ because the actual axial rigidity of the straight tube, *EA*/*L*, is much greater than the effective axial rigidity of the bellows, $k_x$. The geometrical dimensions ($r_{\textrm{m}}$, *t*, *L*, $L_0$) and material constants (*E*, $k_x$) are listed in Supplementary Table S2. Substituting these into Eq. (3), we indeed obtain the ratio of $\gamma ^*/\gamma \approx 24.6$. This rough estimation shows that the bending deformation inside the bellows-type structure takes high priority over the torsional deformation, which is why the alternative pseudo-rotations tend to be induced at the joints. The coupling mode of piecewise deflections and discrete rotations emerges at the onset of a change in conformation.

To investigate the conformational deformation dependent on the number of bodies (*n*), we performed image processing for deformed structures with $n=8, 12$, and 16 in double-traction tests: the structural size for $n = 16$ is a maximum within the allowable deformation under its own gravity. In this experiment, a rotary actuator was used to capture images of the morphology from an arbitrary angle by rotating the object on the horizontal plane; here, the rotation angle is denoted $\theta$. In the initial configuration, the geometry in the image for $\theta =0^\circ$ (i.e., the front view) appears as an imperfect circle because of the force of gravity and the geometric boundary condition—both ends of the loop are fixed perpendicularly to special attachment plates hanging from the actuator. Details of the experimental system are described in Supplementary Information S2.3.

**Figure 3:**
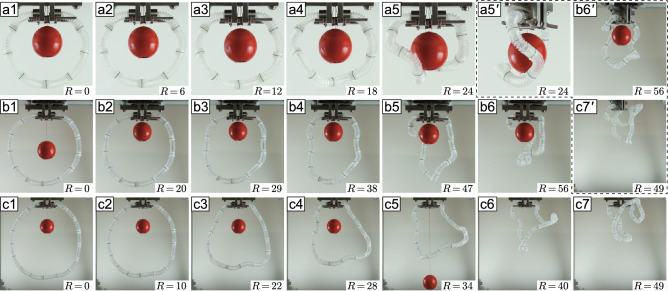
Deformation snapshots for $n=8, 12$, and 16: front views for (**a1**)–(**a5**) $n=8$ at $R=0$–24, (**b1**)–(**b6**) $n=12$ at $R=0$–56, and (**c1**)–(**c7**) $n=16$ at $R=0$–49. Figure (**a5′**), (**b6′**), and (**c7′**) inside the dashed box are the $60^{\circ }$-rotation images of (**a5**), (**b6**), and (**c7**). Note that *R* is the number of rotation actions for the ratchet-type hand winch. In image processing, the overhanging red ball was used to extract the vertical axis of the center, with the string aligning with the rotation axis of the actuator. The indicator was lowered so as not to make contact with the deformed structure.

Figure 3 shows front views of the conformational deformations for (a1)–(a5) $n=8$, (b1)–(b6) $n=12$, and (c1)–(c7) $n=16$. These deformations proceeded with the rotation action of the ratchet-type hand winch, denoted *R* as the action number, where $R=12$ means a full revolution of the winch gear. Figure 3a$5'$,b$6'$,c$7'$ show respectively different views of (a5), (b6), and (c7) at $\theta = 60^{\circ }$.

For the structure with eight segments ($n=8$), the conformational deformation begins at the earlier stage of $R=9$ and forms a wrenched circle at $R=24$ (a5 and a$5'$), being similar to that in Fig. 2e. With $n=12$, a 3D deformation appears at $R=20$ (b2) and undergoes an overall twisting as seen in b3 to b5. The structure then folds from $R=47$ to 56 (b6 and b$6'$). At $n=16$, the deformation of the structure distinctively has two phases. The first is the onset of the conformation at $R=10$, which proceeds along with the counterclockwise rotation of the entire structure as seen in c2 to c5. The structure then folds as the lower half rises (c6 and c7).

To characterize the conformational deformations observed above, we assess the surface area of the convex hull derived from a point set of the positions of joints from the front view of the structure. We first performed a 3D reconstruction of the joint positions in world coordinates (*X*, *Y*, *Z*) from multiple-angle views of the deformed structure within $\theta \in [-60^\circ,60^\circ ]$ by working an ellipse fitting onto blue stripe markers on the joints (see Supplementary Information S3). We computed a series of convex hulls from the given sets of the acquired 2D positions (*X*, *Y*) of the displaced joints during deformation. The convex hull yields the area enveloped by the conformation projected onto the *XY*-plane, which we denote by *S*. The curve of *S*(*R*) provides a criterion for the onset of conformational deformation as demonstrated in the next section for later discussion.

**Figure 4:**
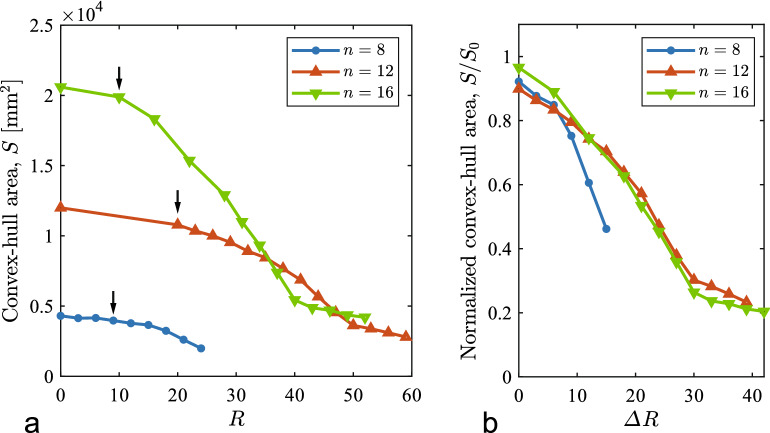
Changes in the convex-hull area during traction tests: (**a**) *S* vs. *R*, in which the downward arrows in (**a**) correspond to onsets of the conformation at $R_{\textrm{cr}}$, with $R_{\textrm{cr}}=9, 20$, and 10 for $n=8, 12$, and 16, respectively—critical points we identified through visual observation; (**b**) $S/S_0$ vs. $\Delta R$, where $S_0$ is the initial value of *S* and $\varDelta R=R-R_{\textrm{cr}}$.

Figure 4a presents the changes in *S* to *R* for $n=8, 12$, and 16. On each curve of *S*(*R*), there is a considerable decrease after the onset of the conformation ($R \equiv R_{\textrm{cr}}$), indicated by a downward arrow; we measured the critical points visually during the experiments. Furthermore, the two curves for $n=12$ and 16 have second inflection points, which correspond to folding. Figure 4b is a modification of the diagram in (a) with the normalization of *S* by the initial convex-hull area $S_0$ and the shift in *R* by $R_{\textrm{cr}}$. In (b), the normalized curves for $n=12$ and 16 agree well with each other regardless of the difference in deformation morphology.

We further examined the results of the double-traction tests for three loops with $n=8$ to elaborate on the 3D reconstruction and the load response of the structure. Supplementary Informations S3 and S4 explain in detail the 3D position estimation of joints and the load measurement device, respectively. Each test was terminated when the traction line was broken at the contact points in the attachment outlet, where the line is bowed the severest (see Fig. S7b).

**Figure 5:**
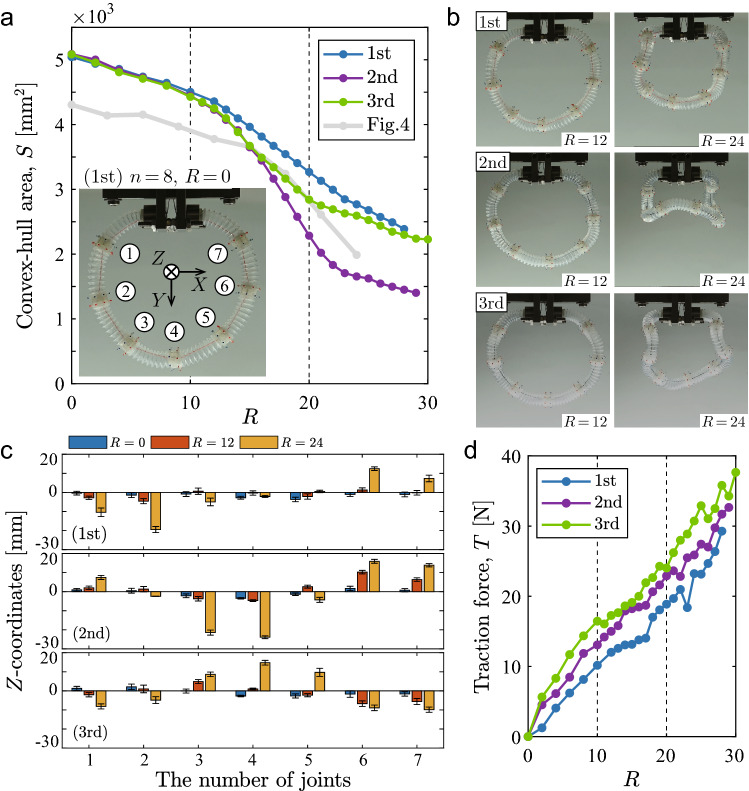
Results for the three loops with $n=8$: (**a**) changes in the convex-hull area, *S*(*R*); (**b**) deformation images at $R=12$ and 24 in each test; (**c**) *Z*-coordinates of joints (blue, red, and yellow bars) at $R=0, 12$, and 24, where the numbers of joints are allocated in a counterclockwise manner from the fixed end [see inset in (**a**)]; (**d**) changes in the force responses, *T*(*R*).

Figure 5a–d presents the experimental results. Figure 5a shows the change in the convex-hull area, *S*(*R*), which is superposed on that in Fig. 4a for $n=8$. For all curves, the decrease in *S* begins with the near-initial value of $S_0$, but the three post-critical responses differ after the onset of the conformation at $R\approx 12$. The second trial path has a steeper slope after the first inflection point in comparison with the first and third trial paths. Additionally, the three curves exhibit a second inflection point around $R = 20$ to 24 to a greater or lesser extent, the nature of which agrees well with the folding modes of the structures with $n = 12$ and 16 (see Fig. 4b). The front views of the three structures at $R = 24$ (Fig. 5b) show different deformation shapes whereas such differences are not clearly distinguishable immediately after the conformation at $R=12$.

We performed the 3D reconstruction of a set of joints in world coordinates (*X*, *Y*, *Z*), with the rotational axis of the actuator aligning with the *Y*-axis and the *XY*-plane being parallel to the camera image at $\theta =0^{\circ }$. The deformed structures at $R = 0, 12$, and 24 are characterized by the *Z*-directional depth positions of joints (see Fig. 5c). The three traction tests yield three different conformation patterns at $R = 24$, which might be affected to no small extent by the initial imperfection of structures at $R = 0$. The *Z*-directional profiles at $R=24$ display antisymmetry in the first test, quasi-symmetry in the second test, and symmetry in the third test. Roughly speaking, the second and third profiles may be inverted by a change in sign, but the second profile has a distinct peak for the third and fourth joints at the bottom, which well represents the folding observed noticeably in the second test (Fig. 5b). The three detailed deformations are discussed with the snapshots in Supplementary Information S5.

The three measured forces (Fig. 5d) exhibit similar increase in response to *R*, although the magnitudes are different. For all curves of *T*(*R*), the structures softened moderately until the onset of the conformation at $R\approx 12$. In the conformational deformation, the slope of *T*(*R*) reduces once but the structures gain resistance. When $R \ge 20$, the traction force *T* drops instantaneously around $R=23$ (first trial), $R=22$ (second trial), and $R=20$ (third trial). This indicates the second inflection point of the curve of *S*(*R*) in Fig. 5a, which may be attributed to a frictional slip with a physical contact between the traction line and inner surface of a tube.

## Discussion

From a mechanical aspect, we next discuss the conformational deformation observed in the double-traction tests. The in-plane deformation up to the onset of conformational deformation can be represented as an even in-plane deformation of a curved beam element that substitutes for the bent bellowed tubes undergoing compression and bending. The details of modeling are described in Supplementary Information S6.

In the initial configuration of a circular loop, a uniform distribution of self-stress induced by its pure bending moment exists along the entire structure. This self-stress is released in response to a traction force during the double-traction test. We consider the state of the bending moment vanishing on all the joints together as the onset of conformational deformation so that the balance of each joint could be satisfied at any configuration of the conformation. Specifically, this hypothesis is a necessary condition for conformational deformation and is adopted latter in performing the convex-hull analyses.

In observing the traction line to be extensible, we set a correction factor $\lambda$ to link a ratchet action *R* to the actual compression displacement *u* of a loop structure. Based on the mechanics of materials, we are led to a linear relation for the traction force *T* with *u* and then derive an equation for *T*(*R*) as in Eq. (S26). To compare with the force responses (see Fig. 5d), the measured traction forces *T*(*R*) are about two times higher than those predicted, which means that in experiments the friction along a line on the joints and the attachment apparatus is non-negligible; see Fig. S21 for details. Adopting the vanishing-moment hypothesis at joints, we find the critical point $(T_{\textrm{cr}}, u_{\textrm{cr}},R_{\textrm{cr}})$ of the system bifurcates along a path towards a conformational deformation.

**Figure 6:**
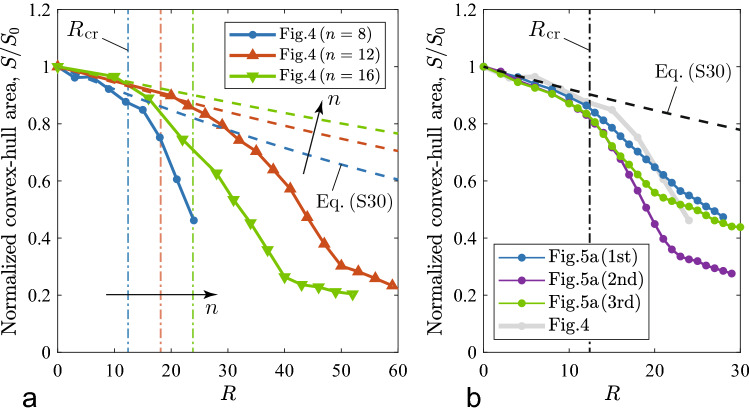
Comparison of $S/S_0$ vs. *R* predictions (dash) obtained from Eq. (S30) with measurements (solid) shown in (**a**) Fig. 4 and (**b**) Fig. 5. In the pre-conformational model, the assumption is that an initial loop with $n+1$ segments, including the attachment space, is contracted uniformly in 2D. A common correction factor of $\lambda \approx 0.1315$ is used; see Sec. S6.3 for details. The vertical lines indicate the critical points of $R_{\textrm{cr}}$ obtained from Eq. (S27).

The pre-conformational model enables us to readily formulate a change in the convex-hull area, *S*(*R*), when considering the isometric shrinkage of the corresponding polygon. Figure 6a,b compare the curves of the normalized convex-hull area, $S/S_0$ between the predictions (dash) and measurements (solid); see Figs. 4 and 5, respectively. Except for $n=16$, the model predicts the pre-conformational deformation well, in that the measured curves deviate from those predicted in the critical zone around $R=R_{\textrm{cr}}$.

The inconsistency for $n=16$ can be explained as follows. In actuality, an inhomogeneous in-plane deformation is caused by an initial imperfection that localizes the internal forces. This localization tends to hasten the onset of a conformational deformation and indeed it can be observed in the initial configuration with $n=16$, being distorted substantially by gravity that acts on it (Fig. 3c1). In other words, this non-uniform distribution of self-stress may cause a local conformation under a small traction force. If deformation proceeds, the tangent stiffness sometimes increases considerably through physical contact from corrugations in bellows. It is the location most likely where a line reaches its tensile strength.

## Concluding remarks

In summary, we proposed a multi-jointed loop structure comprising a finite number of elastic tubular bodies, alternately connected with revolute joints. Subjected to concentrated forces acting toward the center, the structure is capable of forming 3D conformations through the coupling of piecewise continuum deflections and discrete revolute rotations.

Conformational deformations were demonstrated in traction tests of the traction line passing through the loop structure, which depends on the type of traction (single/double traction), the number of bodies (*n*), and the initial imperfections of the structure that lead to phenomena such as buckling. Note that *n* also corresponds to the number of point forces acting on the joints.

The elastic-body geometry also plays an important role in 3D deformation; thus, the bellows-type shape may produce a conformation effect whereas the straight-type may not. In this study, we only focused on the ratio of torsional to bending stiffness, $\gamma = GI_{\textrm{p}}/EI$, determined by the geometric parameters of the tube. From a material parameter perspective, we can tune $\gamma \propto G/E$ to select a material with a high shear coefficient. For example, an anti-torsion slender body may be realized using fiber-reinforced material to optimize the fibrous direction. Alternatively, no upper bound of *G*/*E* exists in continuum mechanics because $G/E = 1/2(1+\nu )$, where $\nu$ denotes the Poisson ratio and $\nu \in [-1,1/2]$ for an isotropic material^31^. An elastic body with a large value of $\gamma$ can potentially be developed using auxetic materials with negative Poisson ratios^31–35^.

Considering a discrete system as an atomistic structure, the three potential energy functions given in terms of bond stretch, bond angle bending, and dihedral angle torsion are respectively equivalent in structural mechanics to the elastic strain energy of stretching, bending, and twisting under infinitesimal deformation^36^. Therefore, the stiffness condition of $\gamma$, needed for conformational deformation, can be readily imposed on the atomistic structures governed by the interatomic interactions of molecular mechanics.

By applying image processing techniques, deformation analyses were performed on 3D reconstructions of the displaced joints. The convex-hull area *S*(*R*) quantifies the conformational deformation projected onto the plane coordinates in front view. There are two inflection points on a single curve of *S*(*R*), the first indicating the onset of the conformational deformation and the second signifying the folding deformation, which does not appear necessarily.

The three structures for $n=8$ with initial imperfections yielded three different morphologies, each final form of which can be represented by the tangle model (see the conformation images in Supplementary Information S5). The conformity is reasonable from the principle of minimum energy; the conformational deformations may be described by the elastic strain energy of the only circumferential normal strain associated with the axial compression and bending of bodies.

Last, we predicted the pre-conformational deformation using the linear analyses with a curved beam element and discussed the differences between predictions and measurements. Both the changes in normalized convex-hull area are consistent at the early stage of deformation and also the onsets of 3D deformation are well estimated given the condition of zero moment acting on joints. Moment-free-joints indicate that self-stresses never exist in the deformed configuration. From energy considerations, therefore, a series of conformations arises as a consequence of exploring the lowest energy configuration of a loop without self-stress, which is the driving mechanism underlying conformational deformation. For a comprehensive understanding of the deformation mechanics with a large set of test pieces, we first need to identify and reduce unexpected frictional forces inside the structure to reconsider the integrated design of the materials and experimental system.

The mechanical stress field in our developed loop structures cannot be translated directly into the real complicated intermolecular forces. However, the measured deformation morphologies correspond to the conformations of the tangle model, which are linked to the conformations of cyclic molecular systems^25^. We therefore expect that mechanical insights into the new deformation phenomena will provide unique perspectives on more complex conformation states of molecular chains and protein architectures^37,38^. Although these deformations behave passively in our experiments, active conformational deformation is also possible from the multi-jointed elastic loop by controlling the unidirectional bending of soft actuators^39,40^. In this manner, friction-free planar rotations work preferentially on joints instead of through disallowed pseudo-rotations.

## Methods

Supplementary methods, materials, figures, tables and discussions are presented in detail in Supplementary Information.

## Supplementary Information

Supplementary Movie S1.

Supplementary Movie S2.

Supplementary Movie S3.

Supplementary Movie S4.

Supplementary Movie S5.

Supplementary Information 6.

## Data Availability

The datasets generated and/or analyzed during the current study are available from the corresponding author (H.T.) on request.
