# Supplementary material for: Conformational deformation of a multi-jointed elastic loop
Source: Sci Rep. 2022 Nov 21;12:19984. doi: 10.1038/s41598-022-24355-7 (PMC9678883; doi:10.1038/s41598-022-24355-7)
Supplement: Supplementary file 6 — Supplementary Information 6. [file 41598_2022_24355_MOESM6_ESM.pdf]

Supplementary Information

Conformational deformation of a multi-jointed elastic loop

Hiro Tanaka[a,*], Yuji Seki[a], Shohei Ueno[a], Yoji Shibutani[a,b]

[a] *Department of Mechanical Engineering, Osaka University, 2-1 Yamadaoka, Suita, Osaka 565-0871, Japan*
[b] *Nanotechnology Program, VNU Vietnam Japan University, Luu Huu Phuoc Street, My Dinh 1 Ward, Nam Tu Liem District, Ha Noi, Viet Nam*

Supplementary Information List:

- Supplementary Information, including Supplementary Figures and Tables.
  - S1 Assembly of small and large multi-jointed loop structures
    - S1.1 Large components
    - S1.2 Small components
  - S2 Setup of experiments and measurements
    - S2.1 Traction test for a large loop
    - S2.2 Comparative traction tests between bellows and straight tubes
    - S2.3 Traction test with shooting deformation
  - S3 Image processing method and data analysis algorithm
    - S3.1 Definitions of coordinate systems
    - S3.2 3D position estimation of joints
    - S3.3 Detection and identification of joint markers
    - S3.4 Convex-hull analysis
  - S4 Load measurement device
  - S5 Discussion on geoemtric symmetry of conformational deformations
  - S6 Discussion on pre-conformational mechanics
    - S6.1 Formulation of infinitesimal in-plane deformation
    - S6.2 Relationship between traction force and the revolution of a winch
    - S6.3 Relationship between convex-hull area and the revolution of a winch
- Caption list for Supplementary Figures S1–S23
- Caption list for Supplementary Tables S1 and S2
- Caption list for Supplementary Movies S1–S5
- References in the Supplementary Information

---

*Corresponding author.
Email address: htanaka@mech.eng.osaka-u.ac.jp (Hiro Tanaka)

S1. Assembly of small and large multi-jointed loop structures

We constructed multi-jointed loop structures in two different sizes. Table S1 provides the material information for all the components, including the segments, joints and traction lines of small or large size (S/L-size).

S1.1. Large components

Figure S1(a) presents the components of the large loop as shown in Fig. 1 of the main text. A single module is assembled with a commercial bellows tube and 3D-printed joints, colored orange, which are fabricated by a 3D-printing machine adopting fused deposition modeling (uPrint® SE Plus, Stratasys Ltd.). In this assembly, the length and outer diameter of a segment are approximately 60 and 14 mm and the height and outer diameter of a joint are 12.45 and 17 mm, respectively.

As shown in Fig. S1(b), the 3D printing model is capable of the one-piece fabrication of a revolute hinge that has two parts (Parts 1 and 2) sharing a common rotation axis. In the two parts, a through hole is set so that a steel wire can pass through without spoiling the function of the revolute pair. A circular trench on the outside face of each part is bonded at each end of a tube using instantly bonding glue. Part 2 has several ventholes for the ejection of soluble support material during support cleaning.

The diameter of a steel wire is 1 mm. We applied a lubricant to the wire.

Table S1: Materials for the prepared components.

Component	Material
Bellows tube (L-size)	Polypropylene (PP)
Bellows tube (S-size)	Perfluoroalkoxy alkanes (PFA)
Straight tube (S-size)	Silicone rubber
Revolute hinge (L-size)	ABS-like resin
Revolute hinge (S-size)	Acrylic UV-curable resin
Traction line (L-size)	Steel wire
Traction line (S-size)	High-strength polyethylene (PE) fibers

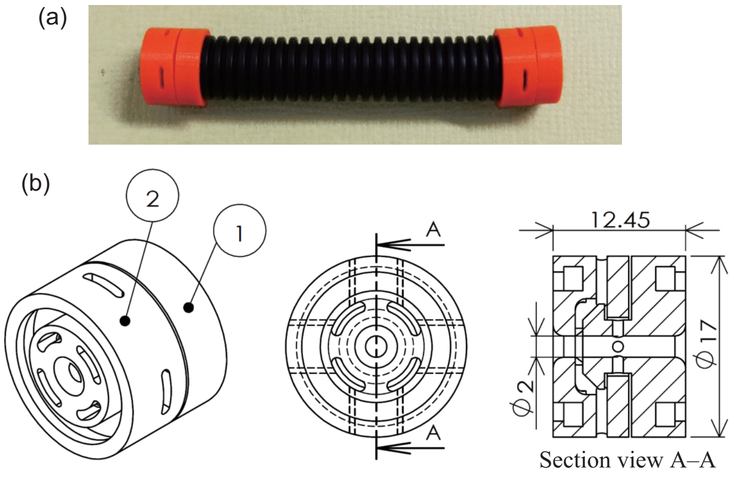

Figure S1: Components of the large loop: (a) bellows tube and revolute hinges; (b) 3D-printed model of the revolute hinge, which comprises two parts (Parts 1 and 2).

S1.2. Small components

For the small loops, we used a commercial flexible tube made of PFA (Penn & Nitto Corp.) as a bellows-type segment. The geometry of the bellows tube is illustrated in Fig. S2, and the dimensions and material properties are listed in Table S2. Note that we calculated the total length of the inclined corrugations, L_0, by representing the corrugations approximately as a sawtooth waveform (see the left inset of Fig. S2). The effective axial rigidity (k_x) of a bellows tube in the longitudinal direction was measured using a horizontal tension tester (SH-14NB, Imada-SS Corp.).

We also prepared a commercial silicone rubber tube as a straight-type segment. The straight tube has outer and inner diameters of 8 and 6 mm respectively; i.e., $r_\mathrm{m} = 3.5$ mm and $t = 2$ mm. The Young modulus of silicone rubber is $E \approx 10$ MPa.

The small joints were fabricated using a 3D printing machine with multiple jets (ProJet 3500 HD Max, 3D systems, Inc.). The material was acrylic ultraviolet (UV)-curable resin. The 3D-printed model (Fig. S3) was roughly half the size of the large model.

We used a commercial fishline produced by Duel Co., Inc., which was easy for us to handle. The braided line was made with high-strength polyethylene fiber (IZANAS®, Toyobo Co., Ltd.). We

Table S2: Dimensions and material properties of the small bellows tube.

Material thickness (t)	0.41 mm
Inner diameter (d_1)	5.5 mm
Outer diameter (d_2)	9.4 mm
Average radius (r_m)	3.73 mm
Segment length (L)	24.8 mm
Total corrugation length (L_0)	39.0 mm
Effective axial rigidity (k_x)	3.3 N/mm
Young's modulus (E)	330 MPa

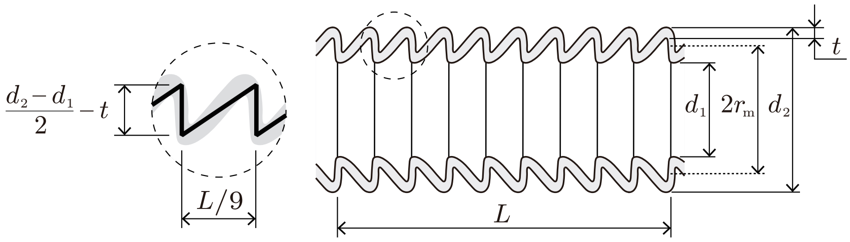

Figure S2: Schematic of the small bellows tube. In the inset, we approximate the corrugations using a sawtooth wave to calculate the total arc length.

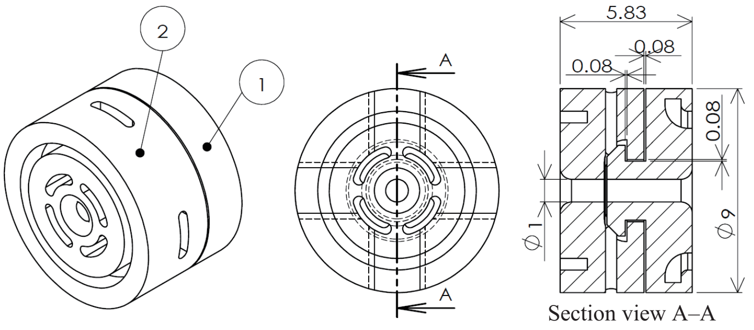

Figure S3: 3D-printed model of a revolute hinge comprising two components (Parts 1 and 2).

applied a lubricant to the traction line.

S2. Setup of experiments and measurements

S2.1. Traction test for the large loop

We conducted traction tests with the large loop assembled as explained in Sec. S1.1. Figure S4 shows an overview of the experiment. In the main body, one joint is replaced with a specially processed pipe fixed on the substrate. The pipe has a slit, from which the steel wire is escaped the structure. One end of the wire is fixed on the substrate and the other end is attached to the hand winch via a roller in the single-traction test (Fig. 1(b)), whereas both ends are attached to the same hand winch via different rollers in the double-traction test (Fig. 1(c)). The hand winch is used for a ratchet function and the single/double-traction test was carried out by rotating the handle of the winch.

S2.2. Comparative traction tests between bellows and straight tubes

We here explain the traction tests as mentioned in Sec. 2 of the main text, which discusses how the shape of segments contributes to the stereoscopic deformation of the multi-jointed loop structure. Figure S5 shows the experiment overview of the double-traction test, where both ends of a non-closed structure were simply constrained by a mechanical vise. By reeling a single line with the same hand winch shown in Fig. S4, we performed the double-traction test for the small loops with bellows and straight tubes.

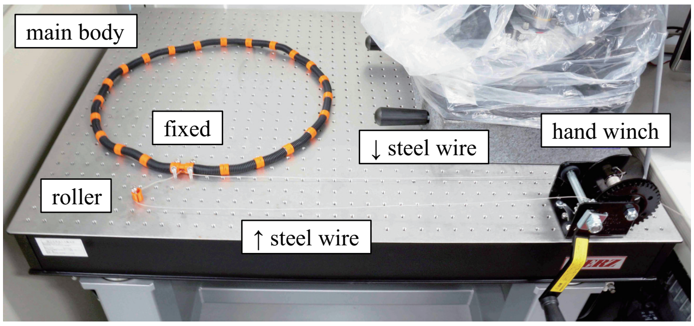

Figure S4: Traction test for the large loop in Figs. 1(b) and (c) of the main text.

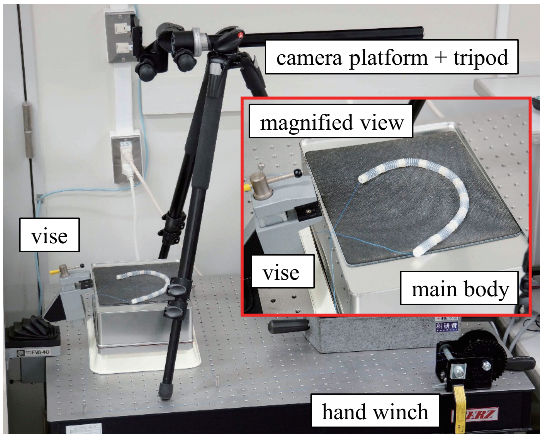

Figure S5: Traction test for the small loop in Fig. 2 of the main text.

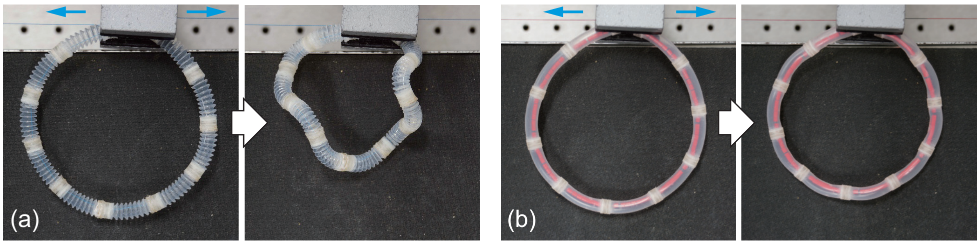

Figure S6: Initial and deformed configurations of the small loops for $n = 10$ in double-traction tests: (a) bellows and (b) straight tube geometries. In (b), the deformation was constrained within the same plane.

Figure S6 shows the initial and deformed configurations for the bellows-type and straight-type structures with $n = 10$. There was conformation for the bellows tube segments (Fig. S6(a)) whereas the structure with straight tubes only contracted in the same plane (Fig. S6(b)). Note that the red plastic beads inside the straight tubes are merely inserted to prevent friction from the direct contact of the traction line with the soft silicone rubber tube during deformation.

S2.3. Traction test with the imaging of deformation

We established an experiment system for conducting a traction test in parallel to shooting the deformed structure by camera. Figure S7(a) shows an overview of the experimental system, which comprises the mechanical apparatuses of a hollow rotary actuator (DG85R-ASAAD, Oriental Motor Co., Ltd.), a specific attachment, and a commercial ratchet-type hand winch. The actuator is fixed on the top panel supported by an aluminum framework.

As seen in Fig. S7(b), the main body of the structure is suspended from the actuator via the specific attachment. The blue traction line passes through in the order of the attachment, actuator, and top panel, and the two traction paths of the line toward the winch follow the orange arrows in Figs. S7(a) and (b). A digital single-lens reflex camera (α7 II, Sony Corp.) is positioned in front of the aluminum framework. By actuating the device, the main body is capable of being rotated about the vertical axis perpendicular to the top panel so that during the test we can take the deformation images from various angles in two dimensions.

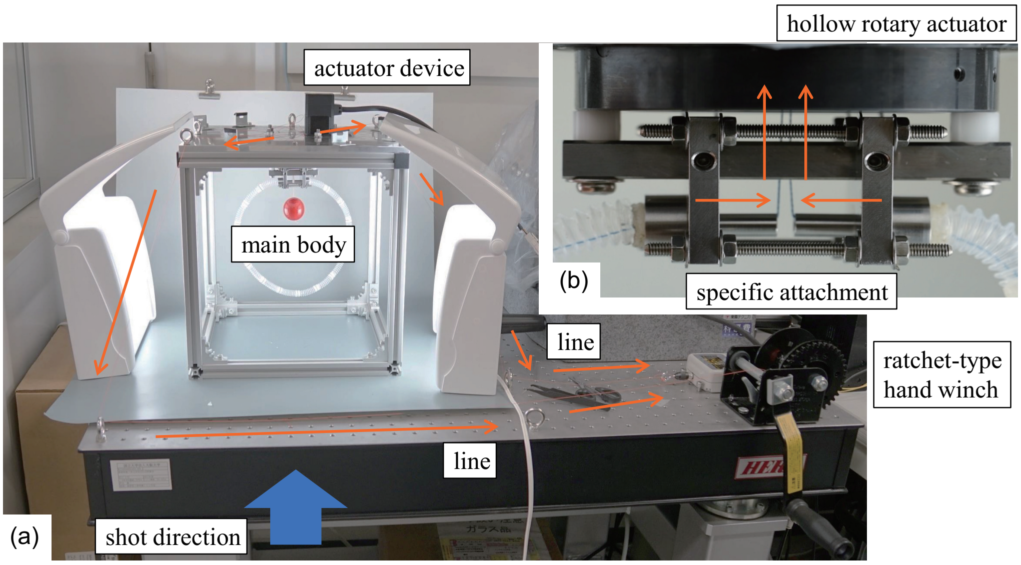

Figure S7: (a) Experiment overview of the double-traction test for the small loop. The orange arrows indicate the traction direction of a single line. (b) Enlarged view of the specific attachment of the structure to the actuator.

S3. Image processing method and data analysis algorithm

S3.1. Definitions of the coordinate systems

We constructed coordinate systems according to camera parameters defined in the Computer Vision System Toolbox of MATLAB®. The camera parameters contain the intrinsic, extrinsic, and lens distortion parameters of a camera.

The intrinsic parameters connect the pixel coordinate system and camera coordinate system. As shown in Fig. S8(a), the pixel coordinates (u, v) can be converted to the image coordinates (x, y) with the optical center (c_x, c_y) of the camera; i.e.,

$$x = \alpha_x(u - c_x), \quad y = \alpha_y(v - c_y), \tag{S1}$$

where α_x and α_y are the x- or y-direction lengths in millimeters per unit pixel and are referred to as conversion ratios. In the perspective projection model (Fig. S8(b)), we denote the camera coordinates by $(X_\mathrm{c}, Y_\mathrm{c}, Z_\mathrm{c})$ and the focal length by f. Because $f : x = Z_\mathrm{c} : X_\mathrm{c}$ and $f : y = Z_\mathrm{c} : Y_\mathrm{c}$, the relationship between the pixel coordinates and camera coordinates is

$$u = f\frac{X_\mathrm{c}}{Z_\mathrm{c}} + c_x, \quad v = f\frac{Y_\mathrm{c}}{Z_\mathrm{c}} + c_y. \tag{S2}$$

In the matrix notation of Eq. (S2),

$$\begin{pmatrix} u \\ v \\ 1 \end{pmatrix} = \boldsymbol{M}_\mathrm{intr} \begin{pmatrix} X_\mathrm{c}/Z_\mathrm{c} \\ Y_\mathrm{c}/Z_\mathrm{c} \\ 1 \end{pmatrix}, \quad \boldsymbol{M}_\mathrm{intr} = \begin{pmatrix} f_x & 0 & c_x \\ 0 & f_y & c_y \\ 0 & 0 & 1 \end{pmatrix}, \tag{S3}$$

where f_x and f_y are respectively the intrinsic x-axial and y-axial focal lengths of the camera. With f, f_x, and f_y, the conversion ratios in Eq. (S1) are determined by $\alpha_x = f/f_x$ and $\alpha_y = f/f_y$. The matrix coefficients $\boldsymbol{M}_\mathrm{intr}$ in Eq. (S3) are referred to as the intrinsic parameters. In each traction test, the respective intrinsic parameters were obtained by running a single-camera calibrator program with checkerboard patterns in the Computer Vision System Toolbox of Matlab® [1].

The extrinsic parameters connect the camera coordinate system and world coordinate system (O-XYZ). In our experiment shown in Fig. S9, the origin of the world coordinate system is located at the center of the rotary actuator. When the actuator is not operated, the undeformed structure

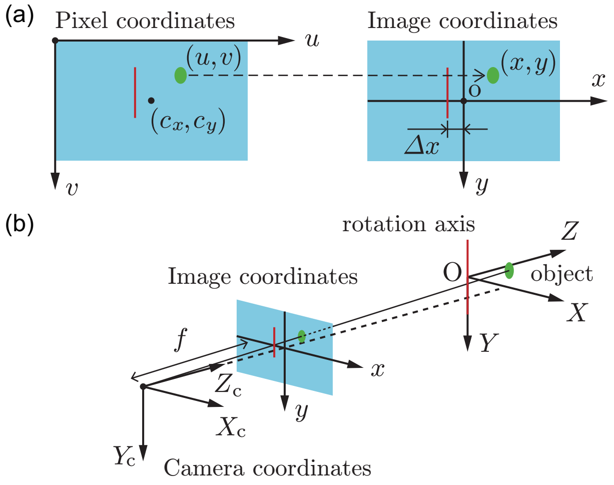

Figure S8: (a) Conversion of the pixel coordinates to the image coordinates. (b) Perspective projection model, where the red vertical line on the Y-axis is the rotation axis of the actuator, extracted from a string suspending a red ball as shown in Fig. 3 of the main text.

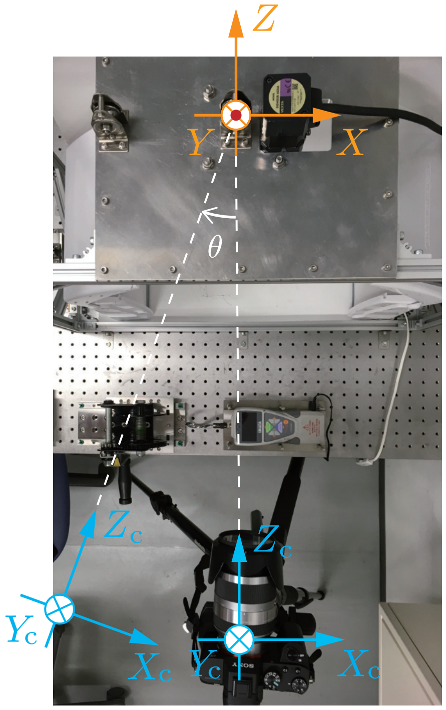

Figure S9: Definitions of coordinates of the experiment system.

appears circular on the image screen. The object of the structure can be shot from an arbitrary angle by rotating the structure on a horizontal plane (XZ-plane) with the operation of the actuator. The rotation angle is denoted θ and is positive in the counter-clockwise direction. The camera coordinate system is then rotated virtually in the clockwise direction by θ around the origin O (see Fig. S9).

The extrinsic parameters $\boldsymbol{M}_{\text{extr}}$ are in general defined by

$$\begin{pmatrix} X_{\text{c}} \\ Y_{\text{c}} \\ Z_{\text{c}} \\ 1 \end{pmatrix} = \boldsymbol{M}_{\text{extr}} \begin{pmatrix} X \\ Y \\ Z \\ 1 \end{pmatrix}, \quad \boldsymbol{M}_{\text{extr}} = \begin{pmatrix} \boldsymbol{R} & \boldsymbol{t} \\ \boldsymbol{o}_3 & 1 \end{pmatrix}, \tag{S4}$$

where $\boldsymbol{R} \in \text{SO}(3)$ is a 3D rotation matrix, $\boldsymbol{t} \in \mathbb{R}^{3\times 1}$ is a translation vector, and $\boldsymbol{o}_3 \in \mathbb{R}^{1\times 3}$ is a zero vector. In the case of our experiment system, $\boldsymbol{R}$ and $\boldsymbol{t}$ are

$$\boldsymbol{R}(\theta) = \begin{pmatrix} \cos\theta & 0 & -\sin\theta \\ 0 & 1 & 0 \\ \sin\theta & 0 & \cos\theta \end{pmatrix}, \quad \boldsymbol{t} = \begin{pmatrix} \Delta x \\ 0 \\ -\ell \end{pmatrix}, \tag{S5}$$

where Δx is the difference between c_x and the x-coordinate of the rotation axis in images, and ℓ is the Z-directional distance of the camera from the origin in the world coordinate system.

S3.2. 3D position estimation of a feature point

We estimated with a single camera the 3D position of an object detected in multiple-angle images by applying the 3D geometric model for a simple stereo system [2]. Figure S10(a) illustrates the initial configuration of the camera geometry under the assumption of a pinhole camera model, including the world coordinate system and camera coordinate system. We denote the positions of the camera and object on the image screen as points A and B. The position vectors of points A and B in the world

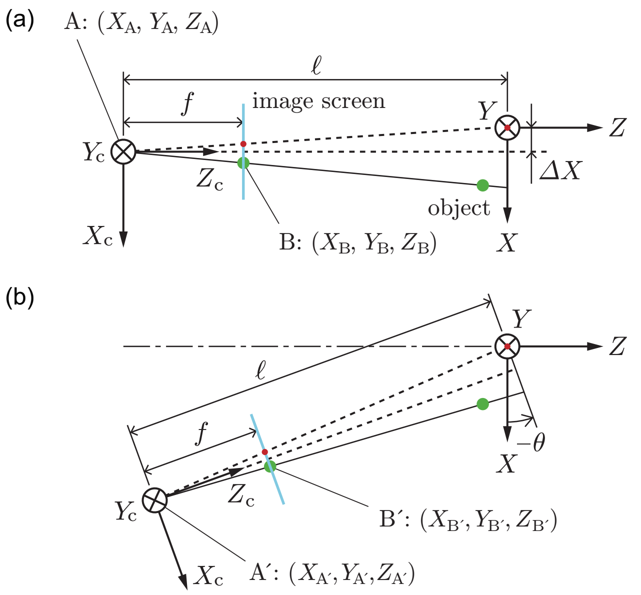

Figure S10: Schematics of the virtual camera geometry: (a) non-operated configuration with $\theta = 0$ and (b) operated configuration with $\theta < 0$. The camera is virtually rotated in the inverse direction of the actuator rotation, θ, being positive in the counter-clockwise rotation; c.f. Fig. S9.

coordinate system are respectively expressed as

$$\boldsymbol{X}_\mathrm{A} \equiv \begin{pmatrix} X_\mathrm{A} \\ Y_\mathrm{A} \\ Z_\mathrm{A} \end{pmatrix} = -\boldsymbol{t}, \quad \boldsymbol{X}_\mathrm{B} \equiv \begin{pmatrix} X_\mathrm{B} \\ Y_\mathrm{B} \\ Z_\mathrm{B} \end{pmatrix} = \begin{pmatrix} \alpha_x(u - c_x) + \Delta X \\ \alpha_y(v - c_y) \\ -\ell + f \end{pmatrix}, \tag{S6}$$

where $\Delta X = \Delta x(\ell/f) = X_\mathrm{A}$. When the rotary actuator is operated with clockwise rotation through θ (< 0), point A can be moved virtually as in Fig. S10(b). The new positions of A and B are denoted A' and B':

$$\boldsymbol{X}_{\mathrm{A}'} \equiv \begin{pmatrix} X_{\mathrm{A}'} \\ Y_{\mathrm{A}'} \\ Z_{\mathrm{A}'} \end{pmatrix} = \boldsymbol{R}^{-1} \begin{pmatrix} X_\mathrm{A} \\ Y_\mathrm{A} \\ Z_\mathrm{A} \end{pmatrix}, \quad \boldsymbol{X}_{\mathrm{B}'} \equiv \begin{pmatrix} X_{\mathrm{B}'} \\ Y_{\mathrm{B}'} \\ Z_{\mathrm{B}'} \end{pmatrix} = \boldsymbol{R}^{-1} \begin{pmatrix} X_\mathrm{B} \\ Y_\mathrm{B} \\ Z_\mathrm{B} \end{pmatrix}. \tag{S7}$$

The parametric equations of lines AB and A$'$B$'$ with $t, s \in \mathbb{R}$ are respectively written as

$$\frac{X - X_\mathrm{A}}{X_\mathrm{B} - X_\mathrm{A}} = \frac{Y - Y_\mathrm{A}}{Y_\mathrm{B} - Y_\mathrm{A}} = \frac{Z - Z_\mathrm{A}}{Z_\mathrm{B} - Z_\mathrm{A}} = t \tag{S8}$$

and

$$\frac{X - X_{\mathrm{A}'}}{X_{\mathrm{B}'} - X_{\mathrm{A}'}} = \frac{Y - Y_{\mathrm{A}'}}{Y_{\mathrm{B}'} - Y_{\mathrm{A}'}} = \frac{Z - Z_{\mathrm{A}'}}{Z_{\mathrm{B}'} - Z_{\mathrm{A}'}} = s. \tag{S9}$$

Ideally, the two lines have an intersection, which is the position of the point object. However, the two lines do not intersect because of several environmental errors. To reconstruct the spatial configuration of the joints, we adopt standard stereo matching. The distance between lines AB and A$'$B$'$ is then given by

$$L = \|t(\boldsymbol{X}_\mathrm{B} - \boldsymbol{X}_\mathrm{A}) + \boldsymbol{X}_\mathrm{A} - s(\boldsymbol{X}_{\mathrm{B}'} - \boldsymbol{X}_{\mathrm{A}'}) - \boldsymbol{X}_{\mathrm{A}'}\|, \tag{S10}$$

where $\|\cdot\|$ indicates the Euclidean norm. To minimize L, we explore t and s such that

$$\frac{\partial L^2}{\partial t} = \frac{\partial L^2}{\partial s} = 0. \tag{S11}$$

By solving t and s in the minimization problem, we find the two points on lines AB and A′B′ that satisfy the shortest distance between the two lines. The midpoint of the acquired points is the estimated position of the object.

S3.3. Detection and identification of joint markers

We adopted two ways of estimating the coordinates of joints by detecting and identifying the joint markers from a set of joint images. In Fig. 3 of the main text, we marked a blue circular strip for each joint (Fig. S11(a)) and estimated the center of the joint by fitting an elliptic function to the marked strip. In Fig. 5 of the main text, we added concentric prickles to a joint (Fig. S11(b)) and estimated the 3D position of the center of the joint from the set of prickle apexes.

Figure S12(a) shows the example of fitting an ellipse to the extracted blue stripe using program code prepared in the Image Processing Toolbox of MATLAB® [3]. In a joint image, an imperfect stripe is obtained because at least half of the stripe is hidden behind the joint object (see Fig. S11(a)). Therefore, the operation of fitting an ellipse to the extracted stripe does not work well. To overcome the difficulty, we first determine the major axis manually and then reflect the imperfect stripe using the major axis in the image coordinate system. Then, using linear least squares fitting [4], an elliptic function is fitted to a given set of points (x, y) including both regions of the originally extracted stripe

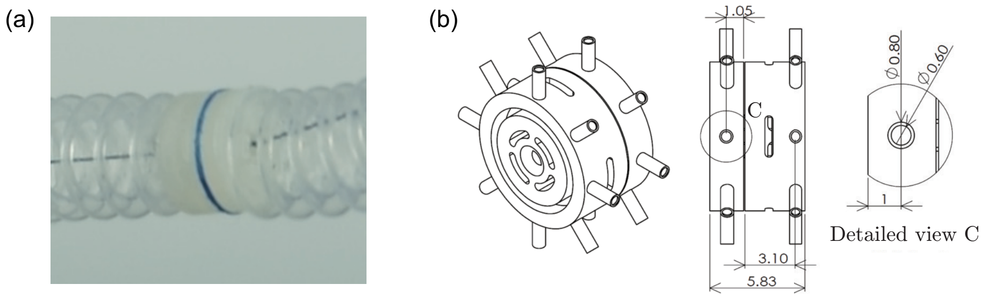

Figure S11: Two types of characteristic marker on a joint for image processing: (a) a blue circular stripe along the perimeter and (b) 3D-printed model with concentric prickles. In the latter case, the dent processed on each apex of prickles is colored to be extracted as a feature point (see the inset of Fig. 5(a) in the main text).

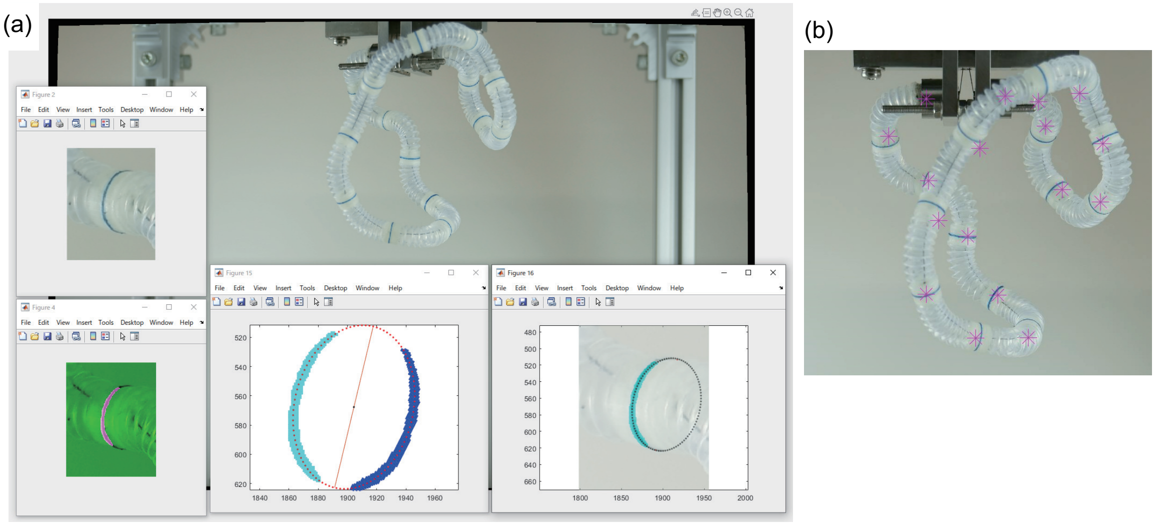

Figure S12: Image processing with regard to Fig 4 in the main text for the structure with $n = 16$ at $R = 49$: (a) a sequence of an ellipse shape extraction of joints, overall rotated by $\theta = -25°$; (b) the estimated positions of joints from the front view of the structure.

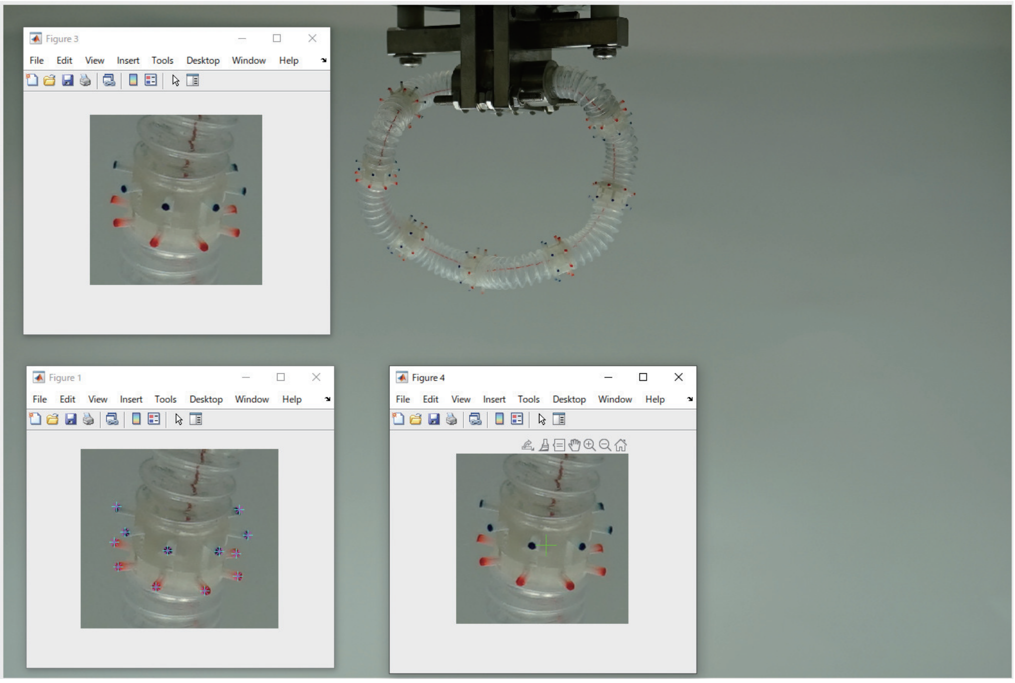

Figure S13: Image processing with regard to Fig. 5 in the main text for the first test at $R = 23$ and $\theta = -25°$. The green cross indicates the centroid of the measured joint.

and reflected stripe (see the inset panel of Fig. S12(a)). The center location $\boldsymbol{p}_j = (p_{xj}, p_{yj})^\mathsf{T}$, semi-major axis a_j, semi-minor axis b_j, and rotation angle α, are thus obtained, where the subscript j stands for the joint number and $j \in \{0, \ldots, n\}$. In each action (R) of the traction tests, we get the ellipse geometry from the joint images by operating the actuator in the range of $\theta = -60°$ to $60°$ at intervals of $5°$. With a set of successful geometric data of the ellipse, $\{\boldsymbol{p}_j^{(i)}, a_j^{(i)}, b_j^{(i)}, \alpha_j^{(i)}\}_{i=1,\ldots,25}$, we estimate the center position of the j-th joint, denoted $\boldsymbol{X}_j = (X_j, Y_j, Z_j)$, by applying the reconstruction method of the multi-view 3D circle [5, 6]. Figure S12(b) presents the example of reconstructed world coordinates (X_j, Y_j) of the deformed structure with $n = 16$ at $R = 49$.

For Fig. 5 of the main text, instead of estimating with the circular stripe on a joint, we adopted the stereo matching method described in Sec. S3.2 by remodeling the small joint with concentric prickles as shown in Fig. S11(b). In the 3D-printed model, two concentric sets of eight prickles are arranged on the upper and lower components. The apexes of the prickles on one side are colored red whereas those on the other side are colored bule. We first extracted the pixel coordinates (u, v) of the apparent apexes on each joint. The center position (u_j, v_j) of a joint was then determined by taking the average of the acquired apex positions. Figure S13 presents the example of detecting the center position of a joint in the first structure with $n = 8$. Within $\theta \in [-80°, 80°]$ at intervals of $5°$, we drove the stereo matching of the corresponding joints between the two rotated structures and reconstructed the world coordinates of the center positions $\{\boldsymbol{X}_j(\theta, \theta')\}_{j=0,\ldots,8}$, where the two parameters indicate a pair of images rotated by θ and θ'. Note that the angle difference of $|\theta - \theta'|$ is greater than $20°$ to assure high accuracy. We first calculated the average $\overline{\boldsymbol{X}}_j$ and standard deviation $\boldsymbol{\sigma}$ from a set of $\{\boldsymbol{X}_j(\theta, \theta')\}$. Generating the subset of $\{\boldsymbol{X}_j(\theta, \theta')\}$ within $\overline{\boldsymbol{X}}_j \pm 2\boldsymbol{\sigma}$ to exclude the outliers, we again calculated the average $\overline{\boldsymbol{X}}'_j$ and standard deviation $\boldsymbol{\sigma}'$. We constructed Fig. 5 in the main text using the updated data $\overline{\boldsymbol{X}}'_j$ and $\boldsymbol{\sigma}'$ as the estimated 3D positions of joints.

S3.4. Convex-hull analysis

With the acquired joint-position set, $\{X_j, Y_j\}_{j=0,\ldots,n}$, for each R, we implemented the convex hull algorithm supported by MATLAB® [7]. The convex hull here means physically the smallest convex

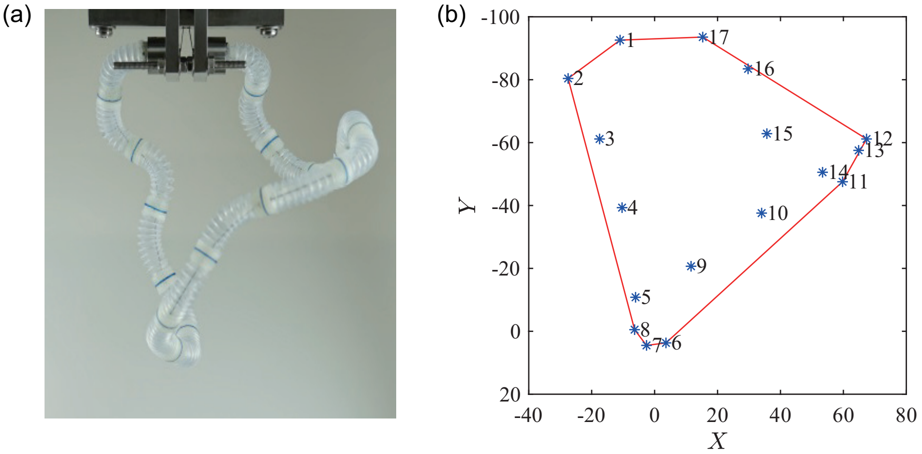

Figure S14: (a) Front image of the structure with $n = 16$ at $R = 40$ and (b) computed convex hull of (a).

polygon that envelopes all the joint positions of a given set, $\{X_j, Y_j\}_{j=0,\ldots,n}$. Figure S14(a) shows the front image of the structure with $n = 16$ at $R = 40$, which is consistent with the image shown in Fig. 3(c6) of the main text. By applying the convex-hull analysis to the deformed structure in Fig. S14(a), we obtained the smallest convex polygon, indicated by red edges, which includes all the joints of blue star markers (see Fig. S14(b)). The convex-hull area S, discussed in the main text, refers to the area of the red convex polygon.

S4. Load measurement device

The force response during the double-traction test is measured using the force measurement device as shown in Figs. S15(a) and (b). We developed the measurement device with a digital force gauge (ZTA-500N, IMADA Co., Ltd.), which is connected via a string to the hand winch that is capable of sliding horizontally on a linear motion guide. During the test, the hand winch is attracted toward the double traction lines and the force gauge fixed on the ground measures the tension acting on the winch.

On the lines from the specimen to the winch, there are several frictional points; i.e., the traction lines inevitably touch the actuator attachment and pulleys. Hence, we carried out the calibration by applying loads of a plurality of weights of 5 N, including a weight table, and the maximum total load

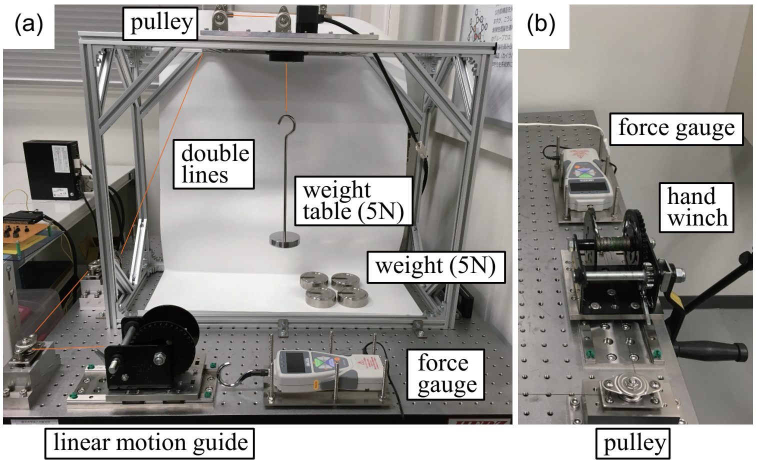

Figure S15: Force measurement system: (a) overview of a calibration test conducted with a digital force gauge and (b) lateral view of the measurement device.

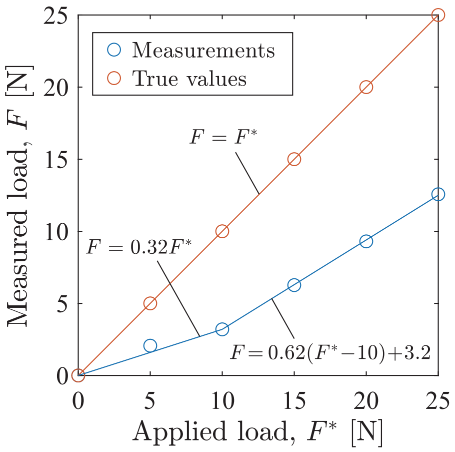

Figure S16: Result of the calibrating tests, where each measured value of F, indicated by a blue circle, is the average of three tests. The blue curve is the piecewise linear function of Eq. (S12), obtained through least-squares fitting.

was 25 N. Figure S16 shows the relationship between the applied load F^* and the load F measured by the force gauge, where each value of F was taken as the mean for three loading tests. The values of F corresponding to $F^* = 5$ and 10 N are more underestimated than those corresponding to $F^* > 10$ N. Therefore, for the correction of the measured load, we used the piecewise linear function

$$\begin{aligned} F &= f_1(F^*) = \frac{b}{10}F^* && \text{if } F^* \leq 10 \text{ N}, \\ F &= f_2(F^*) = a(F^* - 10) + b && \text{if } F^* > 10 \text{ N}. \end{aligned} \tag{S12}$$

With a least-squares method we estimated the coefficients a and b of the second equation of Eq. (S12), resulting in $a \approx 0.62$ and $b \approx 3.2$ N.

In Fig. 5(d) of the main text, the traction forces are corrected by the inverse function of Eq. (S12) as $F^* = f_1^{-1}(F)$ if $F \leq b$ and $F^* = f_2^{-1}(F)$ otherwise. That is,

$$\begin{aligned} f_1^{-1}(F) &= \frac{10F}{b} && \text{if } F \leq b, \\ f_2^{-1}(F) &= \frac{1}{a}(F - b) + 10 && \text{if } F > b. \end{aligned} \tag{S13}$$

In a double-traction test, the traction force T is equal to be $F^*/2$.

S5. Discussion on geoemtric symmetry of conformational deformations

We discuss the conformational deformations of the three different structures for $n = 8$ with the snapshots of front and inclined views shown in Figure S17(a)–(c), which correspond to the three results in Fig. 5(b) of the main text. After the onset of conformations around $R = 10$, these structures evolve their conformations into (a) the pear-shaped form, (b) the folding form, and (c) the chair-like form, respectively. These final morphologies agree well with the conformations of the tangle model as shown in the rightmost insets. The tangle model comprises nine rigid bodies ($n = 8+1$), where the additional black body at top represents a space between the attachment plates in the experiment device. Hence, a little difference in the conformation from the tangle model results from the geometric boundary condition of the right-angled connection to the ceiling actuator.

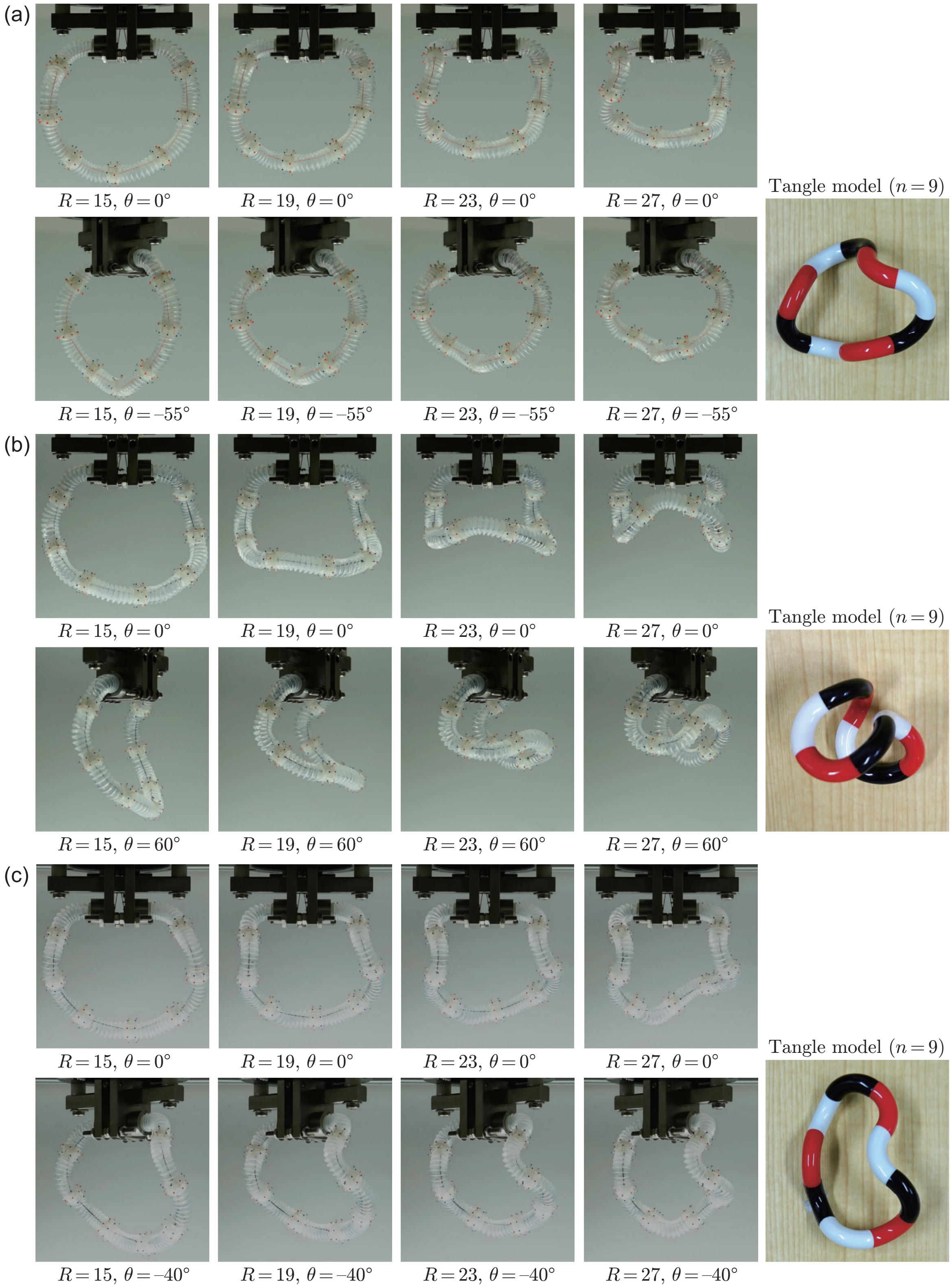

Figure S17: Snapshots of front and inclined views in the conformational deformations for $n = 8$: (a) first, (b) second, and (c) third tests. The rightmost images show the corresponding conformations of the tangle model with $n = 9$, where the black body placed at top is the complement of a space between the attachment plates.

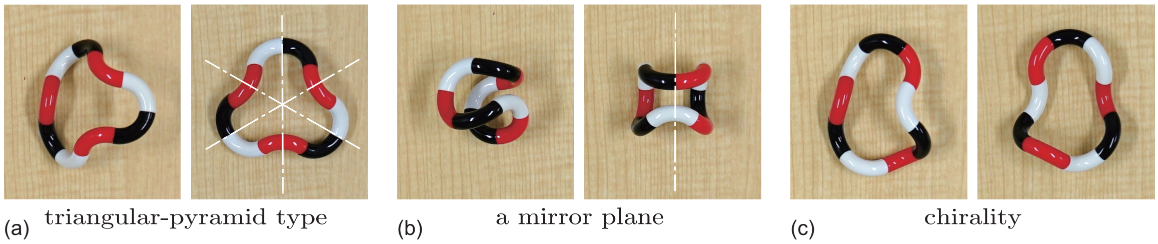

Figure S18: Another-angle-views of the tangle modeds, which are similar with those in Fig. S17(a)–(c), and their geometrical symmetry: (a) $C_{3\text{V}}$, (b) S_1, and (c) C_1.

The three conformations of the tangle model are non-rigid; thus, at least one mechanism exists for each conformation. The first and third states in (a) and (c) belong to a family of non-rigid conformations lying on a two-parameter pathway. Meanwhile, the state of (a) or (c) cannot be converted to (b) without dismantling and reassembling. Figure S18(a)–(c) presents images of a different tangle model corresponding to Fig. S17(a)–(c) to characterize the acquired morphologies of conformational deformation from the geometric-symmetry perspective. Using point group theory, the three conformations are categorized as (a) $C_{3\text{V}}$, (b) S_1, or (c) C_1, where each chain line indicates an improper rotation axis.

In any case, the deformed morphology comprises an axial compression and bending of the elastic bellows without twisting because of the conformation match with the tangle model. In other words, little shear strain is generated in the conformational deformation of our proposed structure. How the deformation proceeds toward the observed conformation is hard to resolve completely at present. However, we can at least state that it is a consequence of exploring the lowest energy configuration without self-stress, an aspect that we shall discuss in the next section.

S6. Discussion on pre-conformational mechanics

S6.1. Formulation of infinitesimal in-plane deformation

To grasp the mechanics of our proposed loop, we formulate the pre-conformational deformation of a simple structural model and predict the onset from an application of linear analysis per segment based on the mechanics of materials. Conveniently, we first adopt the following assumptions:

- Bending of a slender elastic body is subject to Bernoulli–Euler beam theory. The bending is not dominated by shear deformation because of the stiffness properties of bellows as in Eq. (3).
- In a curved beam, the neutral axis in the cross-section is not coincident with the centroid axis [8, 9]. However, we regard the two axes as identical in our beam model, which is valid for a radius of curvature that is sufficiently larger than the cross-sectional dimensions; i.e., the number of segments (n) is preferably large.
- The deformations under compression and bending are mutually independent and occur evenly in all segments. This means that the pre-conformational deformation is sufficient small and the friction of a line on joints is ignored.
- A line from a winch throughout the structure is extensible and the rate of change in the total length of the line inside a shrunk structure is a constant against ratchet actions (R).
- The contact of a line with an elastic body (bellows) is not taken into account during deformation.

Under the above assumptions, let us consider a curved body subjected to traction forces T in a circular loop in a self-equilibrium state (Fig. S19(a)), which is assembled with initially-straight elastic bodies. Because of experimental constraints, the entire loop structure comprises $n + 1$ joints, n elastic bodies, and a single space with the same arc-length L for an elastic body representing the attachment

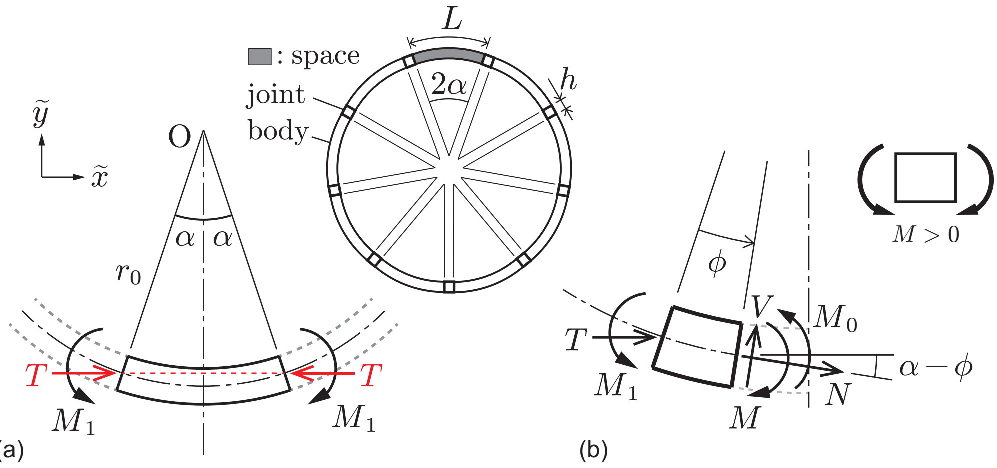

Figure S19: Schematic of an in-plane deformation: (a) self-equilibrium state of an elastic body (in a local coordinate system) subjected to traction forces T at both ends. The inset illustrates a structural model with $n = 8$; (b) free-body-diagram of a section. The inset defines the positive direction of moment.

plates, i.e., $n+1$ elastic bodies. The inset of Fig. S19(a) presents an idealized loop with $n = 8$, where h is the height of a joint. From this structural model, the central angles of a body (2α) and the curvature radius (r_0) are expressed, respectively, by

$$2\alpha = \frac{2\pi}{n+1}, \quad r_0 = \frac{(n+1)L}{2\pi} = \frac{L}{2\alpha}. \tag{S14}$$

According to the free-body diagram (Fig. S19(b)), the dependence of the axial and vertical internal forces, N and V, for a section on angle parameter $\phi \in [0, 2\alpha]$ are

$$N = -T\cos(\alpha - \phi), \quad V = -T\sin(\alpha - \phi), \tag{S15}$$

whereas the internal bending moment M is

$$M = M_1 + M_0 - Tr_0\left(\cos(\alpha - \phi) - \cos\alpha\right), \tag{S16}$$

where M_0 denotes the resultant moment of the self-stresses with respect to pure bending derived from

$$\frac{1}{r_0} = \frac{M_0}{EI^*}. \tag{S17}$$

The complementary strain energy produced by the axial force is expressed as an integral taken along the arc length ($\mathrm{d}s = r_0\mathrm{d}\phi$),

$$U_\mathrm{c} = \int_0^{2\alpha} \frac{N^2}{2EA} r_0 \mathrm{d}\phi. \tag{S18}$$

Substituting Eq. (S15) into Eq. (S18) and using Castigliano's method, the compression displacement u in the direction of T is given by

$$u = \frac{\mathrm{d}U_\mathrm{c}}{\mathrm{d}T} = \int_0^{2\alpha} \frac{N}{EA}\frac{\partial N}{\partial T} r_0 \mathrm{d}\phi = \frac{Tr_0(2\alpha + \sin 2\alpha)}{2k_x L}, \tag{S19}$$

where we recall the effective axial rigidity ($k_x = EA/L$) of the bellows; see Eq. (2).

Denoting the deflection angle by θ, the definition of the change in curvature is $\Delta\kappa = \mathrm{d}\theta/\mathrm{d}s$. We exploit the relation between $\Delta\kappa$ and M and then evaluate the integration from $\phi' = 0$ to $\phi' = \phi$; that

is,

$$-EI^*\Delta\kappa = M \Leftrightarrow -EI^*\theta = \int_0^{\phi} \left[M_1 + M_0 - Tr_0\left(\cos(\alpha-\phi') - \cos\alpha\right)\right] r_0 \mathrm{d}\phi' + C_0$$
$$\Leftrightarrow -EI^*\theta = (M_1+M_0)r_0\phi - Tr_0^2\left(\sin\alpha - \sin(\alpha-\phi) - \phi\cos\alpha\right) + C_0. \quad \text{(S20)}$$

Note that the positive direction of moment is opposite to the curve with $\kappa > 0$ (Fig. S19). For simplicity, the central angle is fixed during the even in-plane deformation of the segments. Then, $C_0 = 0$ in Eq. (S20) from $\theta(0) = 0$. The symmetry of deflection imposes the condition $\theta(\alpha) = 0$, which results in

$$M_1 = -M_0 + Tr_0\left(\frac{\sin\alpha}{\alpha} - \cos\alpha\right). \quad \text{(S21)}$$

Note that the second term is positive for $\alpha \in (0,\pi)$. In Eq. (S21) with $T = 0$, $M_1 = -M_0$ and represents the self-equilibrium state of the initial circular configuration. With Eqs. (S20) and (S21), we have

$$\theta(\phi) = -\frac{Tr_0^2}{EI^*}\left[\left(\frac{\phi}{\alpha} - 1\right)\sin\alpha + \sin(\alpha-\phi)\right]. \quad \text{(S22)}$$

In Eq. (S22), $\theta(2\alpha) = 0$ thereby confirming the symmetry inherent in deflections. Since the rotation of the cross-section is fixed at the three points ($\phi = 0$, α, and 2α), small deformations arising from bending can be ignored.

We give the necessary condition for conformational deformations when considering the balance of a joint. Figure S20(a) shows the free-body diagram of half a joint. The balance condition of moment is expressed by

$$\tilde{M} = M_1 + \frac{Th\sin\alpha}{2}. \quad \text{(S23)}$$

When a conformational deformation occurs, the internal moment $\tilde{M}$ acting on the centroid of the joint should be zero to allow dihedral-angle torsion on the joint (see Fig. S20(b)). Then Eqs. (S17), (S21), and (S23) yield the critical traction force T_{cr} as follows:

$$T_{\mathrm{cr}} = \frac{EI^*}{r_0}\left[r_0\left(\frac{\sin\alpha}{\alpha} - \cos\alpha\right) + \frac{h\sin\alpha}{2}\right]^{-1} = \frac{r_m^2 k_x L}{2r_0}\left[r_0\left(\frac{\sin\alpha}{\alpha} - \cos\alpha\right) + \frac{h\sin\alpha}{2}\right]^{-1}, \quad \text{(S24)}$$

where we use the effective bending stiffness of bellows as in Eq. (2). After the onset of conformation, the 3D deformation may be driven by a couple of non-conservative force vectors of $\boldsymbol{T}$ under an admissible loop configuration from geometry. If the in-plane deformation proceeds through the critical point of $\tilde{M} = 0$, self-stresses return because $\tilde{M} > 0$. In terms of elastic energy, any conformational deformation without self-stress takes priority over the in-plane deformation.

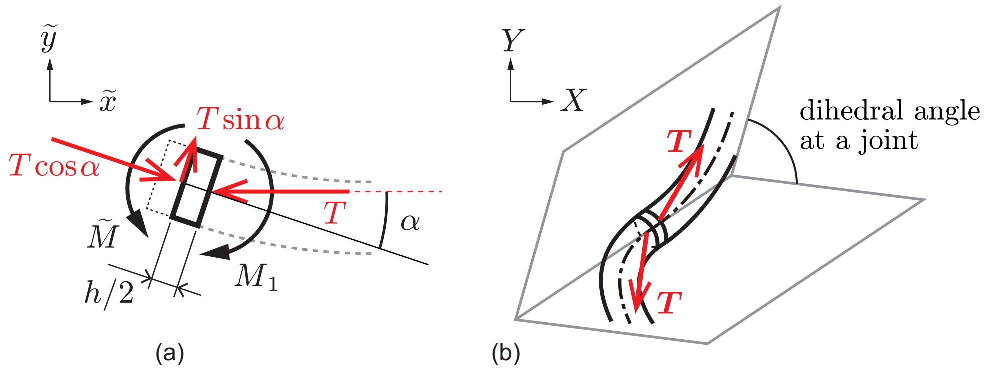

Figure S20: (a) Free-body diagram of half a joint; (b) schematic of the state of conformational deformation.

S6.2. Relationship between traction force and the revolution of a winch

As $R = 12$ indicates a full revolution of the winch gear, the reeling distance per ratchet action in a double-traction test is $\pi d_\mathrm{w} R/6$. We then express the total compression displacement of the structure as a function of R through

$$(n+1)u = \frac{\pi \lambda d_\mathrm{w} R}{6}, \tag{S25}$$

where λ denotes the correction factor required to determine a net change rate in the total length of the extensible line inside the shrunk structure per ratchet action, and d_w the diameter of the winch gear, including the thickness of the reeling-line layer. On substituting Eq. (S25) into Eq. (S19), we find

$$T = \frac{\pi \lambda k_x L d_\mathrm{w} R}{3(n+1) r_0 (2\alpha + \sin 2\alpha)} = \frac{2\pi^2 \lambda k_x d_\mathrm{w} R}{3(n+1)^2 (2\alpha + \sin 2\alpha)}. \tag{S26}$$

With Eqs. (S24)–(S26), the displacement and winch reaction at the critical point are

$$u_\mathrm{cr} = \frac{r_\mathrm{m}^2}{2r_0} G(\alpha), \quad R_\mathrm{cr} = \frac{6 r_\mathrm{m}^2}{\lambda d_\mathrm{w} L} G(\alpha), \quad G(\alpha) = \frac{\alpha(2\alpha + \sin 2\alpha)}{2(\sin\alpha - \alpha\cos\alpha) + (h/r_0)\alpha \sin\alpha}, \tag{S27}$$

where the critical strain, $\varepsilon_\mathrm{cr} = u_\mathrm{cr}/L$, should be within the allowable range for the linear analysis.

To compare with the experiment measurements (Fig. 5(d)), we exploit the relationship between T and R in Eq. (S26), where we use $n = 8$, $L = 24.8$ mm, $h = 5.83$ mm, $d_\mathrm{w} = 20.30$ mm, and $k_x = 3.3$ N/mm (Table S2 and Fig. S3). Figure S21 shows the overlap of the linear equation (S26) on measurements (Fig. 5(d)) for the three tests. Note that each correction factor is calculated using the average changes in measured distance between two adjacent joints from $R = 0$ to $R = 12$; $\lambda = 0.1106 \pm 0.0354$ (the first), $\lambda = 0.1346 \pm 0.0571$ (the second), and $\lambda = 0.1493 \pm 0.0385$ (the third). The measured traction forces are about two times higher than those predicted; the main reason that the linear analyses underestimate $T(R)$ is that the internal friction forces on the attachment components and joints were ignored.

The dashed line in Fig. S21 indicates the level of T_cr calculated from Eq. (S24), which well corresponds to R_cr around the regime of softened T in the measured curves; specifically, $R_\mathrm{cr} \approx 14.75$ (the first), $R_\mathrm{cr} \approx 12.12$ (the second), $R_\mathrm{cr} \approx 10.92$ (the third), and $\varepsilon_\mathrm{cr} \approx 0.0776$ (in all). Considering the

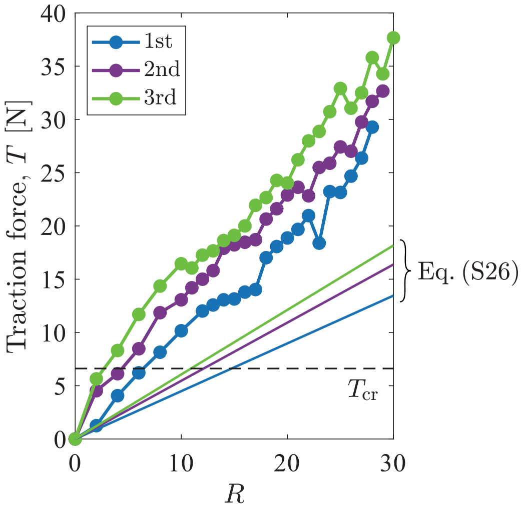

Figure S21: Comparison of F vs. R between the predictions of Eq. (S26) and measurements (see Fig. 5(d)), where we used $n = 8$, $L = 24.8$ mm, $h = 5.83$ mm, $d_\mathrm{w} = 20.30$ mm, $k_x = 3.3$ N/mm (in all data), and $\lambda \approx 0.1106 \pm 0.0354$ (the first), $\lambda \approx 0.1346 \pm 0.0571$ (the second), and $\lambda \approx 0.1493 \pm 0.0385$ (the third).

friction of a line on joints, the deformation is localized at the fixed ends; moreover, the traction forces there are larger than those at any other joint, which hastens the onset of conformation much earlier than that expected from the linear analysis.

S6.3. Relationship between convex-hull area and the revolution of a winch

We read the area of a regular n-gon with side a as

$$S_n = \frac{na^2}{4\tan(\pi/n)}. \tag{S28}$$

During the even progress of in-plane deformations in segments, the change in the side length of a loop is described using Eq. (S25) as

$$a(R) = 2r_0 \sin\alpha + u(R) = 2r_0 \sin\alpha - \frac{\pi\lambda R d_{\mathrm{w}}}{6(n+1)}. \tag{S29}$$

From Eqs. (S28) and (S29), the convex-hull area of a shrinking loop with n segments and an additional single-segment spacing, is obtained from

$$\begin{aligned} S(R) &= S_{n+1}(a(R)) + S_{n+1}(h) + (n+1)r_0 h \\ &= \frac{(n+1)(a^2+h^2)}{4\tan(\pi/(n+1))} + (n+1)r_0 h. \end{aligned} \tag{S30}$$

Note that, in the actual traction test, the length of the attachment space remains unaltered.

Figure S22 shows the changes in (a) convex-hull area $S(R)$ of Eq. (S30) and (b) its normalization by $S(0)$ for a comparison with those of the three traction tests with $n = 8$ (Fig. 5(a)). In Fig. S22(a), the model overestimates the absolute value of $S(R)$ because the attachment space is smaller than the arc length L of the elastic body and the initial loops are distorted. However, the behaviors of the normalized convex-hull area are similar to the measurements taken up to $R = 10$ (Fig. S22(b)). Beyond $R = 10$, the measured curves deviate from the in-plane deformation predictions. Whereas Eq. (S30) is a convex-downward quadratic function of R, the experimental results show that all the curves of $S(R)$ are convex-upward until the secondary inflection points. Hence, the relation $S(R)$ provides a decent criterion for the onset of a conformational deformation. The vertical chain lines of

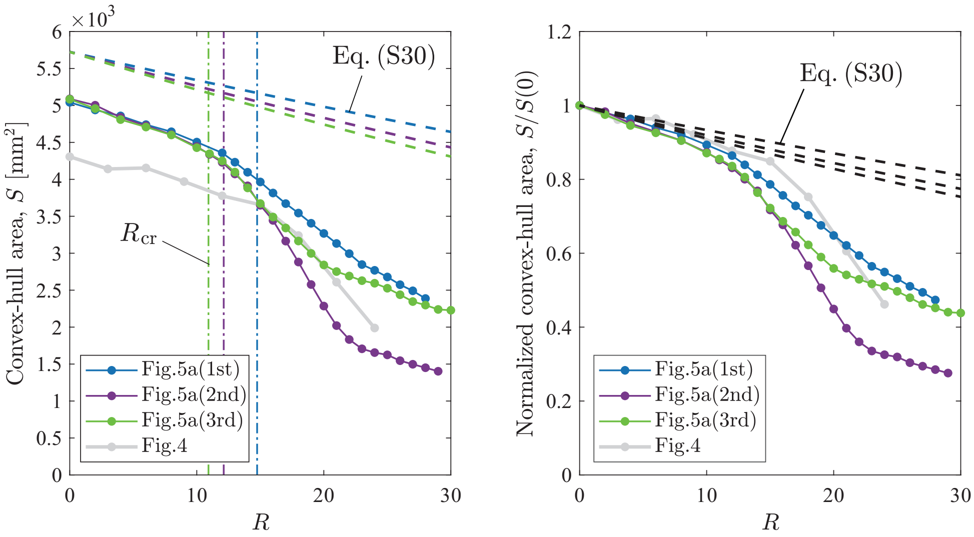

Figure S22: (a) S vs. R and (b) $S/S(0)$ vs. R for $n = 8$; parameter settings are identical to those used in Fig. S21. The numerical curves are superposed on results from Fig. 5(a).

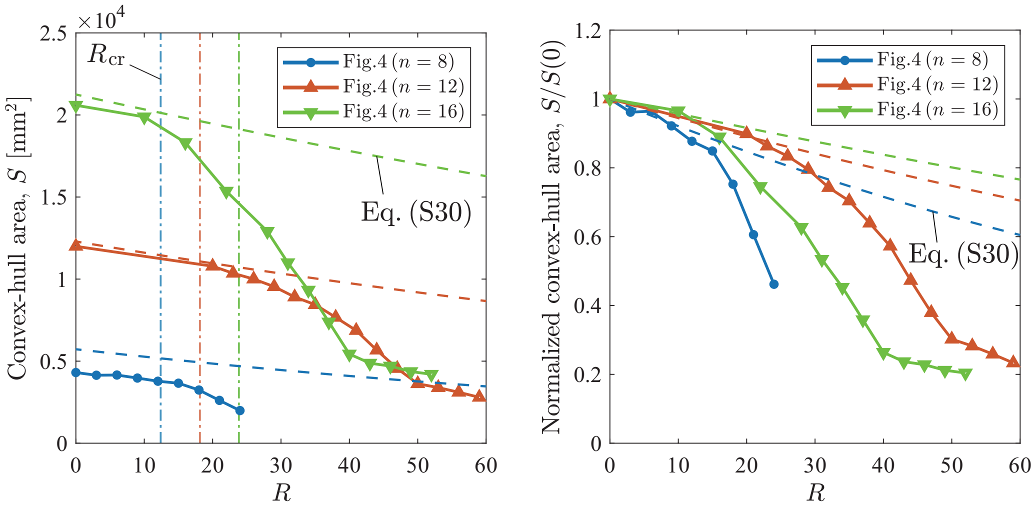

Figure S23: (a) S vs. R and (b) $S/S(0)$ vs R for $n = 8, 12, 16$, where $\bar{\lambda} \approx 0.1315$, which is the average of λ for the three traction tests with $n = 8$. Other parameter settings are identical to those used in Fig. S21. The numerical curves are superposed on results from in Fig. 4.

Fig. S22(a) indicate the critical points of the ratchet actions calculated from Eq. (S27), which agree well with the primary inflection points of $S(R)$ for the three tests.

We also assess the convex-hull area for different numbers of segments. Figures S23(a) and (b) show the changes in $S(R)$ and $S(R)/S(0)$ for $n = 8, 12$, and 16 for a comparison with those in Fig. 4. Note that, as a correction factor, we use the average for λ measured in the three tests with $n = 8$, i.e., $\bar{\lambda} \approx 0.1315$. For $n = 8$ and 12, the two curves of $S(R)$ or $S(R)/S(0)$ well predict the pre-conformational deformations up to $R_{\rm cr}$ indicated by the vertical chain lines. Conversely, the measured $S(R)$ for $n = 16$ drops much earlier than predicted. With $n = 16$, the initial loop has an egg-shaped form when subjected to gravity (Fig. 3(c1)), which is why the non-uniform distribution of self-stress may generate local conformations under the smaller traction force.

Captions for Supplementary Figures S1–S23

Figure S1:

Components of the large loop: (a) bellows tube and revolute hinges; (b) 3D-printed model of the revolute hinge, which comprises two parts (Parts 1 and 2).

Figure S2:

Schematic of the small bellows tube. In the inset, we approximate the corrugations using a sawtooth wave to calculate the total arc length.

Figure S3:

3D-printed model of a revolute hinge comprising two components (Parts 1 and 2).

Figure S4:

Traction test for the large loop in Figs. 1(b) and (c) of the main text.

Figure S5:

Traction test for the small loop in Fig. 2 of the main text.

Figure S6:

Initial and deformed configurations of the small loops for $n = 10$ in double-traction tests: (a) bellows and (b) straight tube geometries. In (b), the deformation was constrained within the same plane.

Figure S7:

(a) Experiment overview of the double-traction test for the small loop. The orange arrows indicate the traction direction of a single line. (b) Enlarged view of the specific attachment of the structure to the actuator.

Figure S8:

(a) Conversion of the pixel coordinates to the image coordinates. (b) Perspective projection model, where the red vertical line on the Y-axis is the rotation axis of the actuator, extracted from a string suspending a red ball as shown in Fig. 3 of the main text.

Figure S9:

Definitions of coordinates of the experiment system.

Figure S10:

Schematics of the virtual camera geometry: (a) non-operated configuration with $\theta = 0$ and (b) operated configuration with $\theta < 0$. The camera is virtually rotated in the inverse direction of the actuator rotation, θ, being positive in the counter-clockwise rotation; c.f. Fig. S9.

Figure S11:

Two types of characteristic marker on a joint for image processing: (a) a blue circular stripe along the perimeter and (b) 3D-printed model with concentric prickles. In the latter case, the dent processed on each apex of prickles is colored to be extracted as a feature point (see the inset of Fig. 5(a) in the main text).

Figure S12:

Image processing with regard to Fig 4 in the main text for the structure with $n = 16$ at $R = 49$: (a) a sequence of an ellipse shape extraction of joints, overall rotated by $\theta = -25°$; (b) the estimated positions of joints from the front view of the structure.

Figure S13:

Image processing with regard to Fig. 5 in the main text for the first test at $R = 23$ and $\theta = -25°$. The green cross indicates the centroid of the measured joint.

Figure S14:

(a) Front image of the structure with $n = 16$ at $R = 40$ and (b) computed convex hull of (a).

Figure S15:

Force measurement system: (a) overview of a calibration test conducted with a digital force gauge and (b) lateral view of the measurement device.

Figure S16:

Result of the calibrating tests, where each measured value of F, indicated by a blue circle, is the average of three tests. The blue curve is the piecewise linear function of Eq. (S12), obtained through least-squares fitting.

Figure S17:

Snapshots of front and inclined views in the conformational deformations for $n = 8$: (a) first, (b) second, and (c) third tests. The rightmost images show the corresponding conformations of the tangle model with $n = 9$, where the black body placed at top is the complement of a space between the attachment plates.

Figure S18:

Another-angle-views of the tangle modeds, which are similar with those in Fig. S17(a)–(c), and their geometrical symmetry: (a) $C_{3\mathrm{V}}$, (b) S_1, and (c) C_1.

Figure S19:

Schematic of an in-plane deformation: (a) self-equilibrium state of an elastic body (in a local coordinate system) subjected to traction forces T at both ends. The inset illustrates a structural model with $n = 8$; (b) free-body-diagram of a section. The inset defines the positive direction of moment.

Figure S20:

(a) Free-body diagram of half a joint; (b) schematic of the state of conformational deformation.

Figure S21:

Comparison of F vs. R between the predictions of Eq. (S26) and measurements (see Fig. 5(d)), where we used $n = 8$, $L = 24.8$ mm, $h = 5.83$ mm, $d_{\mathrm{w}} = 20.30$ mm, $k_x =$ 3.3 N/mm (in all data), and $\lambda \approx 0.1106 \pm 0.0354$ (the first), $\lambda \approx 0.1346 \pm 0.0571$ (the second), and $\lambda \approx 0.1493 \pm 0.0385$ (the third).

Figure S22:

(a) S vs. R and (b) $S/S(0)$ vs. R for $n = 8$; parameter settings are identical to those used in Fig. S21. The numerical curves are superposed on results from Fig. 5(a).

Figure S23:

(a) S vs. R and (b) $S/S(0)$ vs R for $n = 8, 12, 16$, where $\bar{\lambda} \approx 0.1315$, which is the average of λ for the three traction tests with $n = 8$. Other parameter settings are identical to those used in Fig. S21. The numerical curves are superposed on results from in Fig. 4.

Captions for Supplementary Tables S1 and S2

Table S1:

Materials for the prepared components.

Table S2:

Dimensions and material properties of the small bellows tube.

Captions for Supplementary Movies S1–S5

Movie S1:

Animation of a single-traction test for the multi-jointed loop structure with 23 bellows tube segments ($n = 23$) of large size. The traction test was halted at the stress limit of the streel wire.

Movie S2:

Animation of a double-traction test for the multi-jointed loop structure with 23 bellows tube segments ($n = 23$) of large size. The traction test was halted at the stress limit of the streel wire.

Movie S3:

Animation of a double-traction test for the multi-jointed loop structure with 10 bellows tube segments ($n = 10$) of small size.

Movie S4:

Animation of a double-traction test for the multi-jointed loop structure with 10 straight tube segments ($n = 10$) of small size.

Movie S5:
Movies of the unloading of the structure after the double-traction test in Supplementary Movie S3.

References

[1] Matlab ver. R2021b, Single Camera Calibrator App, The MathWorks, Inc., 2021, `https://www.mathworks.com/help/vision/ug/single-camera-calibrator-app.html` (date:06-02-2022).

[2] R. Hartley, A. Zisserman, Multiple View Geometry in Computer Vision, Cambridge University Press (2003).

[3] Matlab ver. R2021b, Get Started with Image Processing Toolbox, The MathWorks, Inc., 2021, `https://www.mathworks.com/help/images/` (date:06-02-2022).

[4] R. Brown, fitellipse.m (matlab program file), `https://www.mathworks.com/matlabcentral/fileexchange/15125-fitellipse-m`, MATLAB Central File Exchange. Retrieved Feburary 6, 2018 (2021).

[5] B. Soheilian, M. Brédif, Multi-view 3d circular target reconstruction with uncertainty analysis, ISPRS Annals of the Photogrammetry, Remote Sensing and Spatial Information Sciences II-3 (2014) 143–148. `doi:10.5194/isprsannals-II-3-143-2014`.

[6] C. Zangl, 3D Circle Reconstruction from Ellipses, Texelography, `http://texelography.com/2019/01/07/3d-circle-reconstruction-from-ellipses/` (date:05-03-2022).

[7] Matlab ver. R2021b, Help center for the “convhull” function, The MathWorks, Inc., 2021, `https://www.mathworks.com/help/matlab/ref/convhull.html` (date:06-02-2022).

[8] W. C. Young, R. G. Budynas, Roark’s Formulas for Stress and Strain, Seventh edition, The McGraw-Hill Companies, Inc. (2002).

[9] Y. Shibutani, A. Nakatani, Mechanics of Materials (in Japanese), Corona Publishing Co., Ltd. (2017).
